# A Win–Loss Interaction on Fe^0^ Between Methanogens and Acetogens From a Climate Lake

**DOI:** 10.3389/fmicb.2021.638282

**Published:** 2021-05-13

**Authors:** Paola Andrea Palacios, Warren Russell Francis, Amelia-Elena Rotaru

**Affiliations:** Nordcee, Department of Biology, University of Southern Denmark, Odense, Denmark

**Keywords:** microbial influenced corrosion, acetogens, methanogens, interspecies interactions, iron corrosion, *Methanobacterium*, *Clostridium*

## Abstract

Diverse physiological groups congregate into environmental corrosive biofilms, yet the interspecies interactions between these corrosive physiological groups are seldom examined. We, therefore, explored Fe^0^-dependent cross-group interactions between acetogens and methanogens from lake sediments. On Fe^0^, acetogens were more corrosive and metabolically active when decoupled from methanogens, whereas methanogens were more metabolically active when coupled with acetogens. This suggests an opportunistic (win–loss) interaction on Fe^0^ between acetogens (loss) and methanogens (win). *Clostridia* and *Methanobacterium* were the major candidates doing acetogenesis and methanogenesis after four transfers (metagenome sequencing) and the only groups detected after 11 transfers (amplicon sequencing) on Fe^0^. Since abiotic H_2_ failed to explain the high metabolic rates on Fe^0^, we examined whether cell exudates (spent media filtrate) promoted the H_2_-evolving reaction on Fe^0^ above abiotic controls. Undeniably, spent media filtrate generated three- to four-fold more H_2_ than abiotic controls, which could be partly explained by thermolabile enzymes and partly by non-thermolabile constituents released by cells. Next, we examined the metagenome for candidate enzymes/shuttles that could catalyze H_2_ evolution from Fe^0^ and found candidate H_2_-evolving hydrogenases and an almost complete pathway for flavin biosynthesis in *Clostridium. Clostridial* ferredoxin-dependent [FeFe]-hydrogenases may be catalyzing the H_2_-evolving reaction on Fe^0^, explaining the significant H_2_ evolved by spent media exposed to Fe^0^. It is typical of *Clostridia* to secrete enzymes and other small molecules for lytic purposes. Here, they may secrete such molecules to enhance their own electron uptake from extracellular electron donors but indirectly make their H_2_-consuming neighbors—*Methanobacterium*—fare five times better in their presence. The particular enzymes and constituents promoting H_2_ evolution from Fe^0^ remain to be determined. However, we postulate that in a static environment like corrosive crust biofilms in lake sediments, less corrosive methanogens like *Methanobacterium* could extend corrosion long after acetogenesis ceased, by exploiting the constituents secreted by acetogens.

## Introduction

Steel infrastructure extends for billions of kilometers below ground enabling not only transport and storage of clean water, chemicals, fuels, and sewage, but also protection for telecommunication and electricity cables. Climate change has led to extreme weather conditions like severe storms and rainfall. Urban storm and rainfall management in many countries, especially northern countries like Denmark, involves so-called climate lakes (also known as stormwater ponds or retention ponds) harvesting rainfall at a large scale, thus alleviating stormwater runoff in the cities (Mishra et al., 2020). If the stormwater runoff is improperly detained, underground steel infrastructure could suffer tremendous damage. Damages induced by microbial-induced corrosion (MIC) underground are often discovered too late, leading to environmental and economic devastation. Thus, it is important to predict the lifespan of the material if exposed to microbial communities native to the site where steel structures are located. This would lead to effective replacement and metal recuperation strategies before accidental spills that may be detrimental to the surrounding environment (Usher et al., 2014a; Skovhus et al., 2017; Arriba-Rodriguez et al., 2018).

In this study, we investigate corrosion by microorganisms from the anoxic sediments of a danish climate lake. In such anoxic environments where non-sulfidic conditions prevail, steel was expected to persist unharmed for centuries (Usher et al., 2014a; Skovhus et al., 2017; Arriba-Rodriguez et al., 2018), and yet, researchers showed that certain groups of anaerobes (methanogens and acetogens) strip electrons off the Fe^0^ surface leading to MIC (Zhang et al., 2003; Mori et al., 2010; Mand et al., 2014, 2016; Kato et al., 2015). Previous studies showed that MIC in non-sulfidic environments is often linked to the presence of acetogens like *Clostridium* and methanogens like *Methanobacterium* or *Methanosarcinales* on the surface of the corroded steel structure (Zhang et al., 2003; Zhu et al., 2003; Mori et al., 2010; Mand et al., 2014, 2016; Kato et al., 2015). It was suggested that *Methanosarcinales* were indirectly involved in corrosion, growing in a mutualistic relationship with the acetogens (Zhang et al., 2003; Mand et al., 2016). This assumption was based on acetogens producing acetate, which acetotrophic *Methanosarcinales* methanogens would then consume. In this case, acetogens were expected to be favored by the removal of their metabolic product—acetate. Such a mutualistic association on Fe^0^ between acetogens and methanogens has not yet been demonstrated experimentally. In contrast, we recently showed that instead of acting cooperatively on Fe^0^, acetogens and methanogens competed for Fe^0^ electrons (Palacios et al., 2019). However, the study by Palacios et al. enriched a corrosive community from a coastal marine environment. In this study, we embarked to understand whether similar interactions apply to corrosive communities enriched from an inland climate lake.

Interspecies interactions on Fe^0^ are scantily examined; here, we investigated the interplay between acetogens (reaction 1) and methanogens (reaction 2) from a climate lake, provided solely with Fe^0^ as their electron donor.

4Fe0+2CO2+4HCO3-+ 4H+→ 4FeCO3+CH3COOH+2H2O(ΔG0′=-388kJ/mol;Reaction 1)4Fe0+CO2+ 4HCO3-+ 4H+→ 4FeCO3+CH4+2H2O(ΔG0′=-446kJ/mol;Reaction 2)

Theoretically, under standard thermodynamic conditions, methanogens should have an advantage over acetogens when provided with Fe^0^ as the sole electron donor (Reactions 1 and 2) especially since methanogens, unlike acetogens, are more effective at retrieving abiotic H_2_ (formed on Fe^0^) due to their low H_2_-uptake thresholds (Kotsyurbenko et al., 2001; Drake et al., 2002). Several groups of methanogens could corrode Fe^0^ independently of acetogenic bacteria, including species of *Methanosarcina* (Daniels et al., 1987; Belay and Daniels, 1990; Boopathy and Daniels, 1991), *Methanobacterium* (Belay and Daniels, 1990; Lorowitz et al., 1992; Dinh et al., 2004), and *Methanococcus* (Mori et al., 2010; Uchiyama et al., 2010; Lienemann et al., 2018; Tsurumaru et al., 2018). The mechanism by which methanogens corrode Fe^0^ has been debated. Some reports suggest that methanogens retrieve H_2_ chemically produced at the Fe^0^ surface (abiotic-H_2_) (Daniels et al., 1987). Others suggest that methanogens retrieve electrons directly using an unknown electron-uptake mechanism (Dinh et al., 2004; Beese-Vasbender et al., 2015). While others showed that certain methanogens produce extracellular enzymes, which catalyze H_2_ evolution at the Fe^0^ surface (Deutzmann et al., 2015; Tsurumaru et al., 2018). The latter mechanism was especially relevant for *Methanococcus* species, which harbored an unstable genomic island encoding [NiFe]-hydrogenases and the heterodisulfide reductase super complex involved in formate generation (Lienemann et al., 2018; Tsurumaru et al., 2018).

Acetogens often dominate corrosive communities, outcompeting methanogens when concentrations of H_2_ are high and temperatures are low, presumably due to the higher kinetics (*V*_max_) of their hydrogenases (Kotsyurbenko et al., 2001). Unlike methanogens, acetogens contain [FeFe]-hydrogenases (Peters et al., 2015), which could retrieve electrons directly from Fe^0^ for proton reduction to H_2_ (Mehanna et al., 2008, 2016; Rouvre and Basseguy, 2016). In this study, we were interested to understand the interspecies dynamics on Fe^0^ between acetogens and methanogens from a climate lake. In the absence of any other terminal electron acceptor but CO_2_, acetogens and methanogens are assumed to be either competing for food (Fe^0^ electrons) or cooperating as outlined below:

1. CO_2_-reducing methanogens (CO_2_ + 8e^–^ + 8H^+^ → CH_4_ + 2H_2_O/CO_2_-reducing methanogenesis) may compete (loss/loss) with CO_2_-reducing acetogens (2CO_2_ + 8e^–^ + 8H^+^ → CH_3_COOH + 2H_2_O/acetogenesis) for Fe^0^ electrons.
2. CO_2_-reducing acetogens (see acetogenesis reaction above) cooperate syntrophically (win/win) with acetoclastic methanogens (CH_3_COOH → CH_4_ + CO_2_/acetoclastic methanogenesis (Zhang et al., 2003; Mand et al., 2016). This type of interaction on Fe^0^ has never been demonstrated experimentally. Conversely, we recently showed that coastal marine *Methanosarcina* competed (loss/loss) with acetogens for Fe^0^ electrons (Palacios et al., 2019).

Here, we used a combination of physiological experiments, inhibition strategies, and molecular analyses to study the acetogens and methanogens from climate lake sediments and disentangle their interactions during Fe^0^ corrosion. In Fe^0^ enrichments from climate lake sediments, we witnessed a new type of interaction (a loss–win interaction) between acetogens and methanogens. Furthermore, physiology experiments combined with metagenomics teased apart the role of acetogens and methanogens in Fe^0^ corrosion and revealed a significant effect of exuded enzymes in promoting H_2_ evolution at the Fe^0^ surface.

## Materials and Methods

### Sample Collection and Enrichment Culture Conditions

Sediment cores were sampled during July 2016 from a small climate lake near a construction site on the University of Southern Denmark Campus, Odense. The salinity of the lake was 0.6 PSU, and gas bubbles (including methane) were continuously released to the water surface while sampling. Sediment cores were sliced in the laboratory. The deeper depth horizon 15–20 cm was used for downstream enrichments. To prepare the original slurries, we used 10 ml of sediment (added with a cutoff syringe) into 50 ml of freshwater media containing 5 g of Fe^0^ granules. An Fe^0^-free control was run alongside. The freshwater media was a modified DSM 120 media (DSMZ, 2014) (modifications: 0.6 g/L NaCl, without casitone, without sodium acetate, without methanol, and without Na_2_S × 9H_2_O). The enrichment cultures were prepared in 50 ml of blue butyl-rubber-stoppered glass vials with an anoxic headspace of a CO_2_:N_2_ gas mix (20:80, v/v). To ensure autotrophic Fe^0^-oxidizing microorganisms became enriched, we incubated strictly with Fe^0^ as the electron donor (for more than 3 years and 11 consecutive transfers). Iron was provided as granules: 1 g/10 ml culture (99.98% Thermo Fisher, Germany) or iron coupons (3 cm × 1 cm × 1 mm).

To examine the stability of the enrichments, methane production was monitored during the first five transfers, each time over the course of 3 months or longer (Supplementary Figure S1). Cultures were stable and produced ca. 3 mM methane per gram of Fe^0^, in all monitored transfers, except for the original slurry, which produced more methane likely due to carry-over organics from the sediment.

When methane production stopped (stationary phase), the enrichments were transferred to fresh media with fresh Fe^0^ granules. Additionally, we monitored methanogen-specific coenzyme F_420_ auto-fluorescence via routine microscopy to confirm the presence of methanogens.

All incubations were performed in triplicate (unless otherwise stated), at room temperature (20°C) in the dark, and without shaking.

**FIGURE 1:**
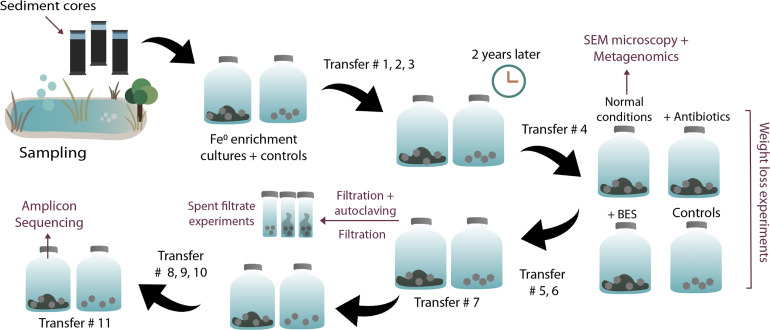
Overview of experimental steps. Enrichments, transfers, and experiments initiated with sediments from a climate lake.

Most downstream experiments (DNA extractions, SEM, inhibition experiments, and end metabolite determinations) were performed at the fourth transfer on Fe^0^ (see Figure 1 of our experimental plan). Shuttle experiments were carried out at the 7th transfer on Fe^0^ and amplicon sequencing at the 11th transfer on Fe^0^.

To evaluate whether methanogens alone are corrosive, we blocked bacterial protein synthesis and bacterial cell wall synthesis with 200 μg/ml of kanamycin and 100 μg/ml of ampicillin, respectively (Palacios et al., 2019). To evaluate the corrosive potential of acetogens alone, we inhibited all methanogens with 2 mM 2-bromoethanesulfonate (BES) (Zhou et al., 2011).

To evaluate the possible role of spent media enzymes/shuttles in H_2_ evolution at the Fe^0^ surface, we withdrew spent media from cultures (at transfer 7) when they have grown on Fe^0^ for 10 days. Ten milliliters of spent media was then filtered and added to fresh and sterile Fe^0^ chips. H_2_ concentration was monitored immediately and after 24 h (*n* = 3; triplicates). In parallel, we also tested autoclaved spent media filtrate (*n* = 3; triplicates), which would inform if the activity was due to the release of enzymes (negatively affected by autoclaving) or released shuttles/corrosive molecules (unaffected by autoclaving). At the same time, we ran abiotic controls (Fe^0^-containing media free of cells; *n* = 3; triplicates) by exposing fresh Fe^0^, to 10 ml “spent” media from abiotic controls incubated for 10 days without cells. Abiotic controls show the extent of H_2_ produced at the Fe^0^ surface in the absence of biological activity and if any chemicals build up even under abiotic conditions to influence Fe^0^ corrosion. Other controls were plain cell filtrate (autoclaved, *n* = 1, and not, *n* = 1) both incubated without Fe^0^, informing whether cellular constituents evolve H_2_ independent of Fe^0^. We detected little to no H_2_ in these control experiments, showing that spent media without Fe^0^ as an electron donor cannot generate H_2_. All spent filtrate experiments were carried out with 10 ml of cell filtrate added to 1 g of fresh Fe^0^ and incubated for 24 h at room temperature. H_2_ monitoring was carried out at the start and after 24 h.

### Chemical Analyses

Methane and hydrogen concentrations were analyzed on a Thermo Scientific Trace 1,300 gas chromatograph system coupled to a thermal conductivity detector (TCD). The injector was operated at 150°C and the detector was operated at 200°C with 1.0 ml/min argon as a reference gas. The oven temperature was constant at 70°C. The separation was done on a TG-BOND Msieve 5A column (Thermo Scientific; 30-m length, 0.53-mm i.d., and 20-μm film thickness) with argon as carrier gas at a flow of 25 ml/min. The GC was controlled and automated by Chromeleon software Dionex, Version 7. In our setup, the limit of detection for H_2_ and CH_4_ was 5 μM.

Acetate production was measured using the Dionex ICS-1500 Ion Chromatography System (ICS-1500) equipped with the AS50 autosampler and an IonPac AS22 column coupled to a conductivity detector (31 mA). We used 4.5 mM Na_2_CO_3_ with 1.4 mM NaHCO_3_ as eluent for the separation of volatile fatty acids. The run was isothermic at 30°C with a flow rate of 1.2 ml/min. The limit of detection for acetate was 0.1 mM.

### Removal of Corrosion Crust and Corrosion Rates

The corrosion crust from the iron coupons was removed with inactivated acid (10% hexamine in 2 M HCl) (Enning and Garrelfs, 2014). Then, the iron coupons were dried with a nitrogen gas stream and weighed.

### Scanning Electron Microscopy

Cells were fixed anaerobically on iron coupons by adding 2.5% glutaraldehyde in 0.1 M phosphate buffer (pH 7.3) and incubating at 4°C for 12 h. The corroded coupons were then washed three times with 0.1 M phosphate buffer at 4°C for 10 min. Dehydration was accomplished by a series of anoxic pure ethanol steps (each step, 10 min; 35, 50, 70, 80, 90, 95, and 100% v/v) (Araujo et al., 2003). The coupons were chemically dried with hexamethyldisilazane under a gentle N_2_ gas stream. Specimens were stored under an N_2_ atmosphere and analyzed within 18–24 h at the UMASS electron microscopy facility using the FEI Magellan 400 XHR-SEM with a resolution of 5 kV.

### DNA Purification From Microbial Enrichments

DNA was extracted from triplicate enrichments grown in 50 ml of media with 5 g of Fe^0^ as the sole electron donor. Extractions were only carried out on the entire corrosive community (untreated with inhibitors) at the 1-month mark of the 4th transfer (for shotgun metagenome sequencing) and 11th transfer (for amplicon sequencing), when both acetogens and methanogens are sufficiently active according to physiology data.

Before metagenome sequencing, genomic DNA was isolated from the pellets of triplicate enrichments (fourth transfer) using commercially available kits, as previously described (Palacios et al., 2019).

Before amplicon sequencing, genomic DNA was isolated from the cell filtrates of triplicate enrichments (11th transfer) using a FastDNA Spin kit for Soil (MP Biomedicals, United States) with the following modifications: to the Lysing Matrix E tube, we added 500 μl of sample, 480 μl of sodium phosphate buffer, and 120 μl of MT buffer. Bead beating was performed at 6 m/s for 4 × 40 s (Albertsen et al., 2015).

The integrity of genomic DNA was verified on an agarose gel and quantified on a mySPEC spectrophotometer (VWR^®^/Germany) or Qubit dsDNA HS/BR Assay kit (Thermo Fisher Scientific, United States).

### Whole Shotgun Metagenome Sequencing, Assembly, and Analyses

DNA from triplicate biological replicates was pooled before whole metagenome sequencing. Sequencing was carried out commercially on a NovaSeq 6000 system, using an Illumina TrueSeq with a single PCR step (Macrogen/Europe). Unassembled DNA sequences were merged, quality checked, and annotated using the Metagenomics Rapid Annotation (MG-RAST) server (v4.03) with default parameters (Meyer et al., 2008). Illumina True Seq sequencing resulted in 3,723,388 high-quality reads of a total of 4,032,354 with an average length of 249 ± 35 bp (Supplementary Table S1). We compared the metagenomic data with the RefSeq database (Tatusova et al., 2015) available on the MG-RAST platform. The alpha diversity for this shotgun metagenome was 61 species. The rarefaction curve indicated that we recovered most of the prokaryotic diversity in this sample (Supplementary Figure S2). To investigate functional genes in the unassembled shotgun metagenome, sequencing reads were annotated against the KEGG Orthology (KO) reference database. Both taxonomic and functional analyses were performed with the following cutoff parameters: *e*-value of 1e^–5^, a minimum identity of 80%, and a maximum alignment length of 15 bp. The unassembled metagenome dataset is available at MG-RAST with this ID: mgm4827981.3.

Before assembling the metagenome, data quality and kmer abundance were estimated using the method of Eitel et al. (2018). The Python and R source code for these steps are available online at https://github.com/wrf/lavaLampPlot. As distinct GC-coverage peaks were clearly visible in the kmer plot, the data were of sufficient quality to continue the assembly. The metagenome was then assembled with MetaSPAdes, a package of the SPAdes v3.14.1 assembler optimized for metagenomes (Nurk et al., 2017), using default parameters. This resulted in 84742 contigs with a contig N50 of 6.7 kb. Most contigs were short and had low coverage, although the largest contig was 489,000 bp. When restricted to contigs over 500 bp, we obtained only 29,455 contigs with a contig N50 of 12 kb, but nonetheless, these accounted for 85% of the assembly.

Binning of the contigs was done manually, using the top BLAST hits to the RefSeq database for each contig, as well as the GC content and coverage. This yielded 10 bins, corresponding to 12 species. Bins for the two predicted archaea were filtered to include only contigs where the best BLAST hit was another archaeon. Proteins were then predicted for all contigs using Prodigal V2.6.3 (Hyatt et al., 2010), using the metagenome mode (option “-p meta”). This gave 1,63,458 predicted proteins in total. Protein counts for each bin are included in the Supplementary Material, Supplementary Table S2. Pathway annotation was done using the KEGG web server BLASTKOALA (Kanehisa et al., 2016) for each bin, selecting “Prokaryotic” annotation mode, using “Genus-Prokaryotes” as the database. Annotations were predicted for >46% of proteins across all bins (see Supplementary Table S2).

All contigs were then uploaded to the MG-RAST server. For the assembled shotgun metagenome, functional gene screening was run on the MG-RAST server in the same fashion as for the unassembled reads (see above). Functional gene screening was also done against the bins annotated with BLASTKOALA. The assembled metagenome dataset is available at MG-RAST with this ID: mgm4916968.3. For the metagenome assembly and annotation, intermediate analyses, code, and commands can be found at https://bitbucket.org/wrf/corrosion-community-2021.

Additionally, we screened the metagenome for hydrogenases by searching for sequence hits with high similarity to those of known [FeFe]-hydrogenases. The resulting 77 genes were then verified for the presence of hydrogenase domains against the most recent CDD/SPARKLE (Conserved Domain Database) database (Marchler-Bauer and Bryant, 2004; Lu et al., 2020) using the batch-blast CD search function on the NCBI platform. At this step, we discarded queries that had not been matched with the hydrogenase superfamily. To conclusively resolve the hydrogenase class, we used the web-based hydrogenase classifier HydDB (Søndergaard et al., 2016).

### Amplicon Sequencing and Analyses

DNA from triplicate enrichments (transfer 11) was sequenced commercially (DNASense ApS/Denmark). The 16S rRNA variable gene regions V4 (Bacteria and Archaea) and V3–V5 (Archaea) were amplified. We did not use the standard Illumina primers because they are less successful at retrieving some Archaeal phyla (Brandt and Albertsen, 2018). Therefore, we used a modified primer pair for the V4 region [515FB] GTGYCAGCMGCCGCGGTAA and [806RB] GGACTACNVGGGTWTCTAAT (Apprill et al., 2015), which is better suited for the detection of most Bacteria and Archaea phyla (Brandt and Albertsen, 2018). Additionally, we also amplified the V3–V5 region using archaeal specific primers [Arch−340F] CCCTAHGGGGYGCASCA and [Arch−915R] GWGCYCCCCCGYCAATTC (Pinto and Raskin, 2012).

The amplicons were then primed for sequencing by the addition of adapters. Then, we sequenced pair-end for V4 (2 × 300 bp) and single-end for V3–5 (1 × 300 bp) on an Illumina MiSeq (Illumina, United States) using a MiSeq Reagent kit v3 (Illumina, United States) according to the Illumina protocol (Illumina, 2015). Negative controls (from DNA extraction and PCR amplification) were sequenced alongside samples. As sequencing control, a PhiX control library was spiked in (at 10%) to overcome issues often noticed with low diversity amplicon samples. All raw metagenomic and amplicon sequencing data have been deposited at NCBI under BioProject accession PRJNA713576.

Forward single-end reads for the V3–V5 variable region were cut to 225 bp. All sequence reads (for V3–5 and V4 variable regions) were trimmed, dereplicated, formatted, and chimera filtered with Trimmomatic v. 0.32 (Bolger et al., 2014) using settings SLIDINGWINDOW:5:3 and MINLEN: 225. For the V4 variable region, which was pair-end sequenced, the forward and reverse reads were merged with FLASH v. 1.2.7, settings −m 10 −M 250 (Magoè and Salzberg, 2011). Trimmed and merged reads were processed using the UPARSE workflow, which has better accuracy and generates realistic OTU counts (Edgar, 2013). The dereplicated reads were clustered, with default settings. OTU abundances were estimated using the settings −id 0.97 −maxaccepts 0 −maxrejects 0. Taxonomy was assigned using the RDP classifier (Wang et al., 2007) in QIIME, –confidence 0.8 (Caporaso et al., 2010) and the SILVA database vs. 132 (Quast et al., 2013). The results were analyzed via the Rstudio IDE using the ampvis package v.2.6.4 (Albertsen et al., 2015).

### Phylogenetic Tree Construction

Phylogenetic trees were constructed in GENEIOUS (Kearse et al., 2012). The closest related sequences (from type cultures or environmental samples) were identified via the BLASTn (for 16S rRNA gene) or BLASTp (for functional genes) against the RefSeq database at NCBI. Sequences were downloaded as FASTA files and imported to GENEIOUS. Sequences were aligned with MUSCLE (Edgar, 2004) using eight iterations, measuring the distance with kmer 6_6 (iteration 1) and subsequently with pctid_kimura (seven iterations), clustering with UPGMB, and with CLUSTALW for sequence weighting, with - anchor spacing 32, – min. length 24. The protein alignment was then manually verified and refined with MAFFT (Katoh et al., 2002; Katoh and Standley, 2013) using - FFT-NS-I ×1,000 algorithm, the BLOSUM62 scoring matrix, and - gap penalty 1.53. The 16S gene sequence alignments were manually verified and refined with MAFFT using – FFT-NS-I x1000 algorithm, the 200PAM scoring matrix, and – gap penalty 1.53.

Trees were constructed with the GENEIOUS Tree Builder using the Jukes-Cantor genetic distance model, and the Neighbor Joining tree building method with 100 bootstraps (support threshold 50%).

## Results and Discussion

### Pitted Fe^0^ Corrosion

We monitored corrosion by a climate lake community transferred only with Fe^0^ as the electron donor over the course of 3 years. The original slurry amended with Fe^0^ generated more methane than a slurry without Fe^0^ (Supplementary Figure S1). Afterward, cultures were transferred (10% transfer) 11 times in fresh media containing only Fe^0^ as the electron donor. After 11 transfers, cultures continued to generate methane from Fe^0^.

**FIGURE 2:**
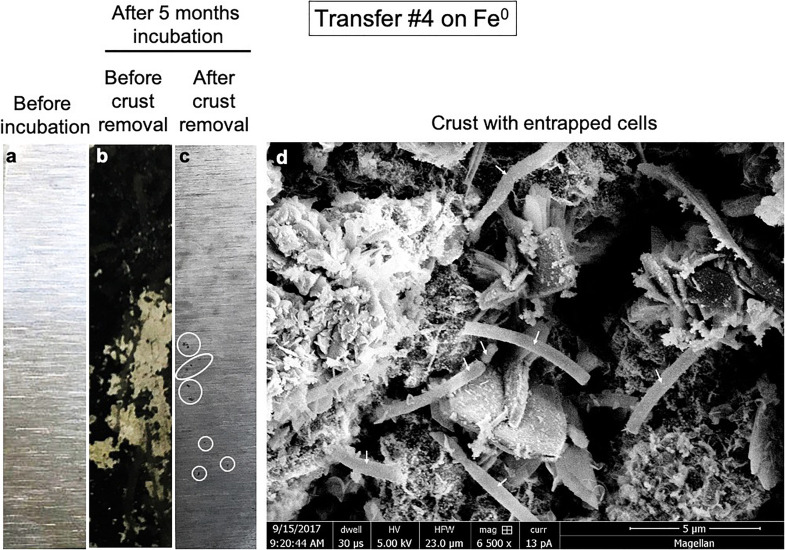
Pitted corrosion by microorganisms. Metallic iron sheets before exposure to cells **(a)**, before crust removal **(b)**, and after crust removal **(c)**. **(d)** A scanning electron micrograph showing cells entrenched on the metal surface.

**FIGURE 3:**
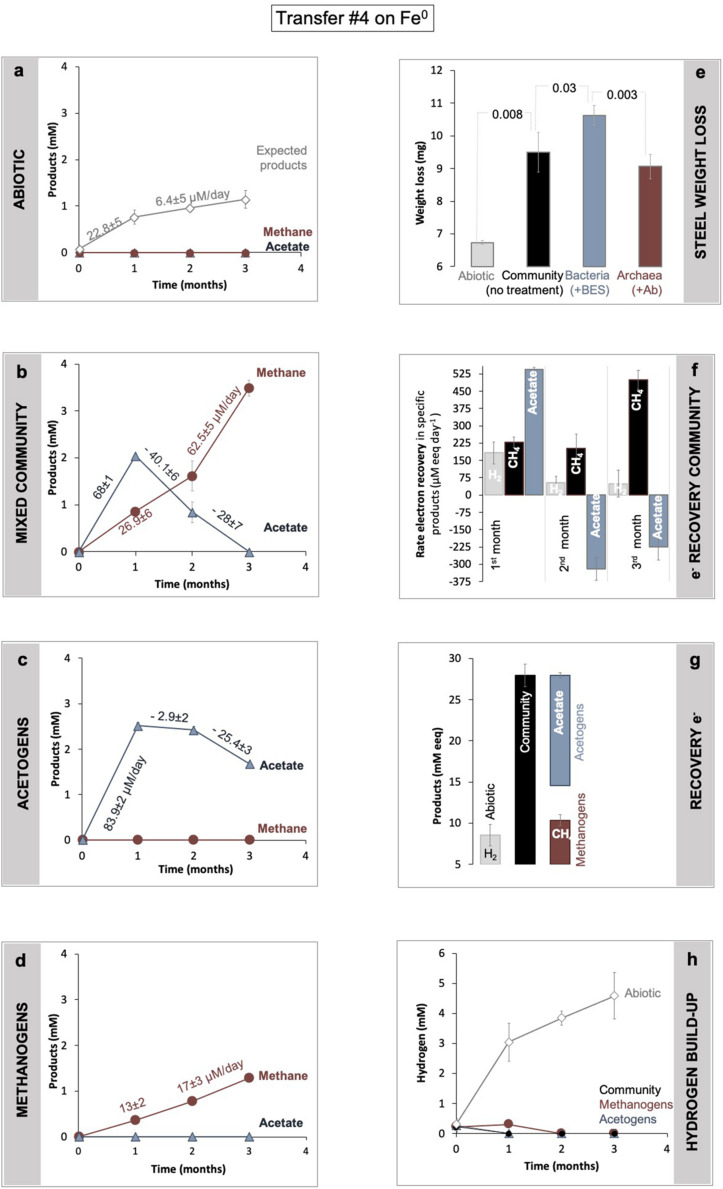
Determination of Fe^0^ corrosion following product formation **(a–d)** and weight loss **(d)**. Product formation was monitored in **(a)** abiotic controls, versus **(b)** a microbial community from SDU sediments **(c)** by bacteria alone, after specific inhibition of the methanogens with 2-bromoethanesulfonate (BES), and **(d)** methanogens alone after inhibition of all bacteria using a mix of antibiotics (Ab). **(e)** Gravimetric determination of material loss under all four conditions (abiotic, with the mixed community, with acetogens alone, with methanogens alone). For the same incubations, panel **(f)** shows changes in electron recovery rates over the course of 3 months and **(g)** total electron recovery into products as mM electron equivalents (eeq) produced from Fe^0^ under all four different conditions. To calculate electron recoveries, we consider 2 mM electron equivalents/eeq per mol H_2_ (according to: 2e^–^ + 2H^+^ → H_2_) and 8 mM eeq for each mol of methane or acetate (according to: CO_2_ + 8e^–^ + 8H^+^ → CH_4_ + 2H_2_O; and 2CO_2_ + 8e^–^ + 8H^+^ → C_2_H_4_O_2_ + 2H_2_O). **(h)** Hydrogen evolution in abiotic controls (mineral media controls) with Fe^0^, versus incubations with cells (community, acetogens, and methanogens alone) and Fe^0^. All incubations were run in parallel and in triplicate (*n* = 3).

We monitored a full corrosion time course after circa 2 years, during the fourth transfer on Fe^0^. The microbial community corroded Fe^0^ significantly in contrast to cell-free controls according to microscopy observations (Figure 2), gravimetric measurements (Figure 3), and metabolic product buildup (Figure 3).

Iron sheets incubated for several months with cells from a climate lake developed a black crust. Scanning electron microscopy revealed that the black crust incorporated long blunt-end rod cells encrusted on Fe^0^ (Figure 2d). The removal of the black crust revealed pitted corrosion underneath (Figure 2).

The lake community induced 41% more Fe^0^ weight loss, consuming 2.8 ± 0.6 mg more Fe^0^ (*n* = 3; *p* = 0.008) than cell-free controls (Figure 3), according to gravimetric measurements.

Metabolic product buildup showed that the microbial community generated methane and acetate simultaneously once provided with Fe^0^ (Figure 3), confirming that Fe^0^ delivers electrons for two types of microbial metabolisms, acetogenesis and methanogenesis. As expected, the abiotic controls with Fe^0^ showed no traces of microbial metabolic products (methane and acetate) (Figure 3).

During a 3-month-long incubation with Fe^0^, the corrosive community formed acetate transiently (first month) and ultimately accumulated only methane (Figure 3). During the first month of incubation, acetogenesis rates (68 ± 1 μM acetate/day) surpassed methanogenesis rates (27 ± 6 μM methane/day) (Figure 3), whereas during the last two months of incubation, acetogenesis ceased. At the same time, methanogenesis sped up, achieving rates two-fold (62.5 ± 5.1 μM methane/day) above those predicted (28 ± 7.3 μM methane/day) by acetoclastic methanogenesis (Figure 3). These results show that methanogens did not rely on the acetate generated by acetogens for methanogenesis. Altogether, the microbial community made 3.3-fold more methane (3.5 ± 0.1 mM) than expected (1.1 ± 0.2 mM) from the H_2_ evolved abiotically (2e^–^ + 2H^+^ → H_2_) at the Fe^0^ surface (Figure 3). These results show that the microbial community employs effective alternative mechanisms (other than abiotic H_2_) to access electrons from the Fe^0^ surface. Unraveling these mechanisms is difficult without pure cultures. Nevertheless, we attempted to determine how the microorganisms influence each other’s metabolism and Fe^0^ corrosion by separating each physiologic group with group-specific inhibitors.

### Unraveling Interspecies Interactions on Fe^0^

In all our enrichments, methane is the final product of the microbial community provided with Fe^0^ as electron donor and CO_2_ as the electron acceptor. Thus, methanogens must interact with acetogens, for example, by consuming their metabolic product. Three possible interspecies interactions would ultimately give off only methane:

(a) mutualism (win/win) when methanogens feed on the product of the acetogen, both partners being influenced favorably, one (acetogen) by the removal of metabolic product and the other (methanogen) by the availability of a food substrate.
(b) commensalism (0/win) when acetogens are unaffected by the presence of the methanogens, whereas methanogens are influenced favorably, e.g., by feeding on acetate, the product of acetogenic metabolism.
(c) parasitism/scavenging opportunism (loss/win) when acetogens are negatively influenced by the presence of the methanogens, while methanogens are influenced favorably by the presence of the acetogens.

To test these scenarios, we carried out inhibition experiments to specifically block each metabolic group. Archaeal methanogens were inhibited with 2-bromoethane sulfonate (BES), a coenzyme M analog (Zhou et al., 2011), resulting in favorable conditions for acetogens. Acetogens were inhibited by a cocktail of antibiotics (kanamycin and ampicillin), thus favoring only methanogens. Then, we compared corrosion by each group alone by documenting corrosion via gravimetric measurements and metabolite production (Figure 3).

According to gravimetric measurements, when separated, methanogens and acetogens remained significantly more corrosive than cell-free controls (Figure 3). However, methanogens were only slightly less corrosive than the mixed community (ca. 5% less, *n* = 3, *p* = 0.18), whereas acetogens were significantly more corrosive alone (ca. 16% more; *n* = 3, *p* = 0.03), denoting that electron uptake from Fe^0^ by acetogens was negatively affected by the presence of methanogens. Furthermore, when examining acetate metabolism, the acetate buildup was faster when acetogens were alone (Figure 3c, 23% rate increase, *n* = 3, *p* = 0.0003) than with methanogens. Thus, the acetogens were more corrosive alone and metabolically better off than within the mixed community (Figure 3). In other words, these results reflect that acetogens are negatively impacted (loss) by the presence of methanogens.

Methanogens, on the other hand, produced three-fold less methane alone than within the mixed community (Figure 3d, *n* = 3, *p* = 0.0002). Methanogenesis was overall faster in the presence of acetogens than when methanogens were incubated alone (Figure 3). Nevertheless, the rates of methanogenesis could not be linked to acetate utilization because acetate consumption (28 ± 7 μM/day) did not match methane production (62.5 ± 5 μM/day). This suggests that methanogens are favored (win) by the presence of the acetogens exclusive of acetate released by acetogens.

Altogether, our results show that during Fe^0^ corrosion, acetogens were negatively (loss) impacted by the presence of methanogens, whereas methanogens were positively (win) impacted, demonstrating a parasitic/opportunistic (loss/win) type of interaction between these two physiologic groups.

**FIGURE 4:**
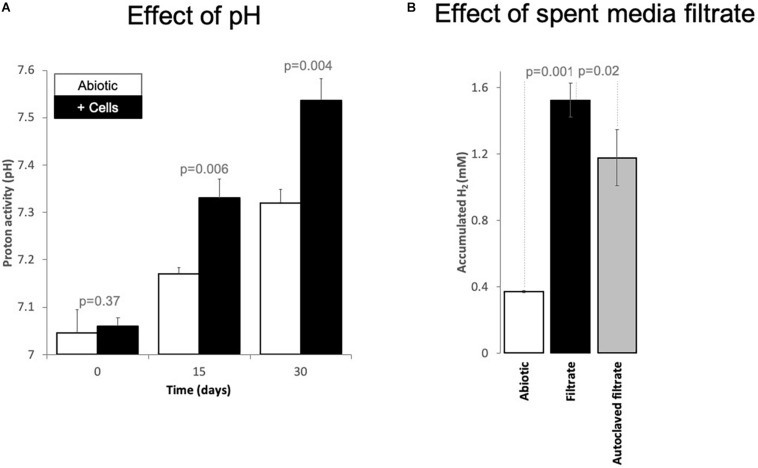
Changes of **(A)** proton activity (pH) and **(B)** H_2_ evolution from Fe^0^ catalyzed by spent media components. **(A)** pH changes in abiotic incubations (white bars, mineral media controls) versus incubations with cells (black bars, mixed corrosive community) exposed to Fe^0^ for 1 month. **(B)** Changes in H_2_ evolution from Fe^0^ when the material got exposed for 24 h to 100% spent media, filtered fresh from a 10-day old Fe^0^-grown mixed community. Additionally, we exposed Fe^0^ to spent media from the same culture that has been inactivated by autoclaving. Also, we tested whether 10-day old abiotic spent media induces any changes in H_2_ evolution from Fe^0^. All experiments were carried out in parallel and in triplicate (*n* = 3). Spent media (autoclaved/not) without Fe^0^ did not produce H_2_ at all (data not shown, values under the detection limit, *n* = 1 for each).

A possible negative effect on the acetogens could be due to the alkalinization of the media by protons being dislocated from solution during CO_2_ conversion to methane. During CO_2_ conversion by methanogens, the pH often becomes alkaline (Xu et al., 2014). Consequently, we verified the pH change over time. Typically, our cultivation media has a pH of 7.06 ± 0.02. However, after 6 months of incubation, four cultures incubated solely with Fe^0^ exhibited an alkaline pH of 8.47 ± 0.06. To verify whether alkalinization was dependent on cellular activity, we monitored pH changes in abiotic Fe^0^ media versus Fe^0^ media with cells (transfer 10) over the course of 30 days. We noticed a significantly higher alkalinization in media with cells than media without cells starting with day 15 (Figure 4). Typically, CO_2_-reducing acetogenesis is negatively affected by alkaline conditions (Mohanakrishna et al., 2015), explaining the decrease in acetogenic activity after 1 month. On the other hand, alkaline conditions inhibit acetotrophic methanogenesis while favoring CO_2_-reducing methanogenesis (Phelps and Zeikus, 1984).

A possible positive effect on the methanogens could be due to H_2_-evolving enzymes being released by acetogens, which reach stationary phase earlier than methanogens. We, therefore, explored whether cell exudates increase H_2_ buildup from Fe^0^. For this purpose, we exposed fresh Fe^0^ to spent media filtrate and compared H_2_ evolution to that observed with the abiotic filtrate. Ten-day-old spent media filtrate stimulated H_2_ evolution four-fold compared to abiotic controls (Figure 4). Acetate cannot catalyze H_2_ evolution from Fe^0^. Thus, the rise of H_2_ buildup by spent media may be due to catalytic molecules (enzymes/non-proteinaceous catalysts, e.g., FeS centers) being released in the media by aged cells, thereby promoting electron capture from Fe^0^ and consequently catalyze H_2_ buildup. Heat treatment of the spent filtrate led to lower H_2_ evolution from Fe^0^, although still three-fold higher than abiotic controls (Figure 4). These results suggest that enzymes have a role in enhancing H_2_ evolution from Fe^0^. However, other undefined spent media components are also evidently involved.

In fact, previous studies revealed that H_2_ evolution could be catalyzed by non-viable cell components (peptides, trace metals), which concentrate, for example, on the surface of electrodes (poised at −600 mV and used as sole electron donor) (Yates et al., 2014).

In our system, the viable media filtrate may contain [FeFe]-hydrogenase and nitrogenase enzymes, which are among the most prevalent enzymes catalyzing proton reduction for H_2_- production. The non-viable cell components in the spent media filtrate could be catalytic active centers and other organic matter-bound redox-active metal centers.

### Corrosive Acetogens and Methanogens

To further investigate the possible interplay between acetogens and methanogens, we investigated the microbial community and their possible metabolic interplay by shotgun metagenomics and amplicon sequencing.

**FIGURE 5:**
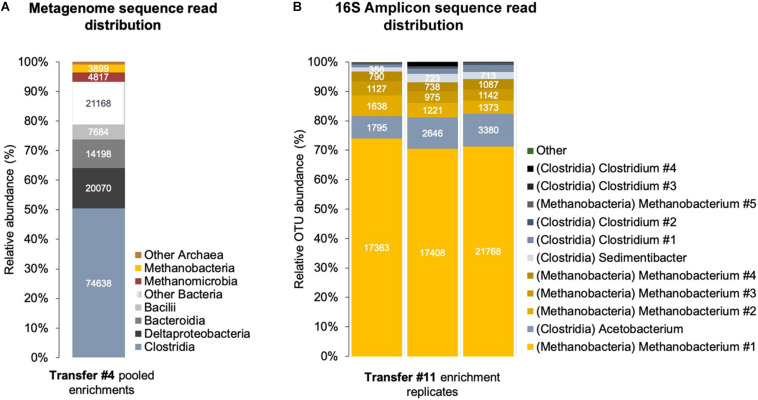
Corrosive microbes from a climate lake enrichment. **(A)** Relative abundance of classes of Bacteria and Archaea in a shotgun metagenome obtained from a sedimentary lake community transferred four times on Fe^0^. **(B)** Relative abundance of OTUs (operational taxonomic units) identified by amplicon sequencing of the V4 region of the 16S rRNA gene in triplicate enrichments.

*Clostridia* and *Methanobacteria* were enriched with Fe^0^/CO_2_ as sole energy sources (Figure 5). We obtained a shotgun metagenome after four transfers when other microorganisms like *Bacteroidetes*, *Deltaproteobacteria*, and *Methanomicrobia* also accompanied the groups above. However, after 11 transfers on Fe^0^/CO_2_ -media, only *Clostridia* and *Methanobacteria* persisted (Figure 5).

Sequential transfers with Fe^0^ under CO_2_-reducing conditions adapted a community with only two C1-based respiratory metabolisms: CO_2_-reducing acetogenesis and CO_2_-reducing methanogenesis. This was apparent in the metagenome from the relative distribution of energy metabolism genes, including C1-carbon metabolism.

#### Acetogens

At the fourth transfer, we identified several acetogenic genera (Supplementary Table S2) by shotgun metagenomics. The class with the highest relative read abundance was *Clostridia* (50.5% of assembled prokaryotic reads), followed by *Deltaproteobacteria* (13.6% of assembled prokaryotic reads) and *Bacteroidia/Parabacteroidia* (9.6% of assembled prokaryotic reads).

**FIGURE 6:**
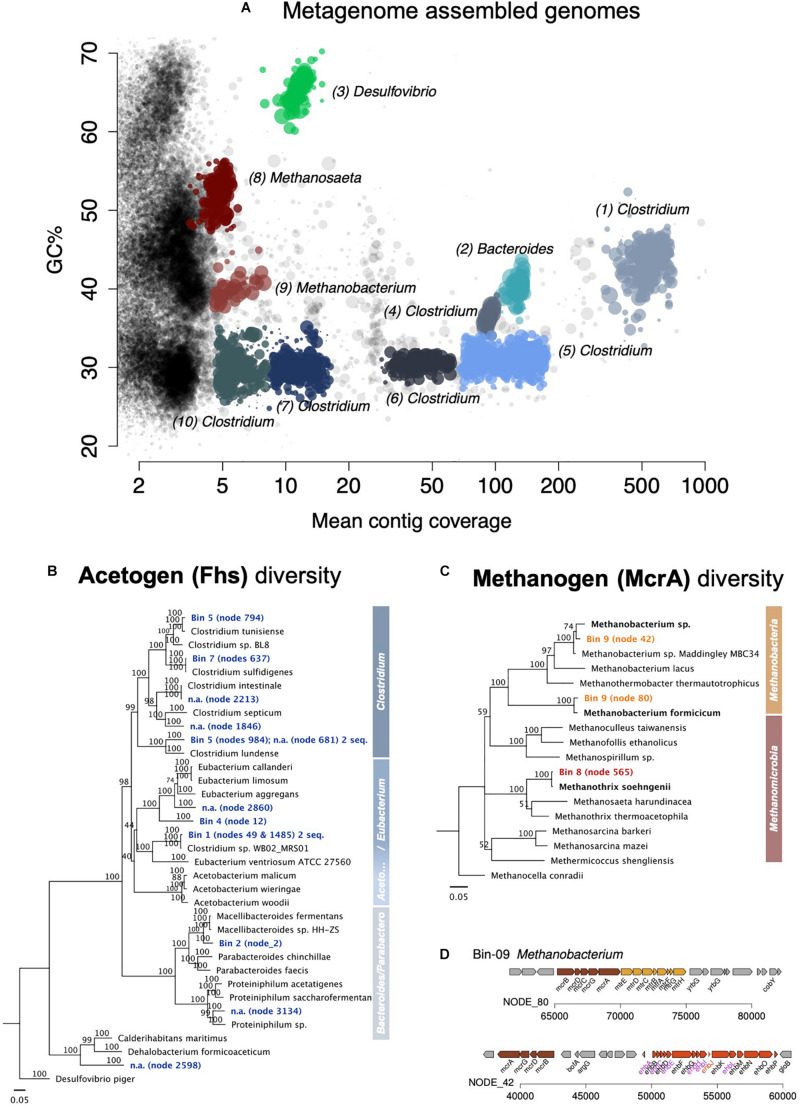
Assembled metagenome bins and the phylogenetic distribution of key genes for acetogenesis and methanogenesis. **(A)** Overview of all assembled metagenome bins in accordance with the GC content and mean contig coverage. **(B)** Neighbor Joining tree depicting the diversity of the key protein for acetogenesis Fhs (formate—tetrahydrofolate ligase). The Fhs of a *Deltaproteobacteria*, *Desulfovibrio piger*, was used as an outgroup root for this tree. **(C)** Neighbor Joining tree depicting the diversity of the key protein for methanogenesis McrA (the alpha subunit of the methyl:coenzyme M reductase). The McrA of *Methanocella conradii* was used as an outgroup root for the tree. Only complete gene sequences were used for the amino acid alignments. A scale of 0.05 means 5% distance. Node numbers show bootstrap values. Values over 70 should be considered significant and supporting the branching point. **(D)** Two complete mcr operons found in the *Methanobacterium*-bin (no. 9).

Eight bacterial metagenomes were assembled into bins or metagenome-assembled genomes and then assigned by MG-RAST to one species of *Desulfovibrio*, one species of *Parabacteroides*, and six or eight *Clostridium* species since some bins appear to contain two different strains (Supplementary Table S2). Analyses of the key gene for acetogenesis *fhs*—formate-tetrahydrofolate ligase (Müller and Frerichs, 2013)—confirmed that five of the bins were inherent acetogens (Figure 6, Supplementary Figure S5, and Supplementary Table S3), four *Clostridium* and one *Parabacteroides*. The *Parabacteroides fhs* was related (93.2% amino acid identity) to that of an uncharacterized *Macellibacteroides* isolate (Supplementary Table S3).

Of the *Clostridium* bins, only two showed high *fhs*-amino acid identity to actual *Clostridium* species in culture (Supplementary Table S3). *Clostridium* bin five included two *fhs*, both with high sequence similarity with the *fhs* in *Clostridium* species, one with 99.5% to *C. tunisiense*, the other 99.2% to *C. lundense*. Also, *Clostridium* bin seven carried two *fhs*, one 99.8% identical in amino acid residues to *C. sulfidigenes*, and the other 90.6% identical to the *fhs* of a *Sedimentibacter*. Nevertheless, the best represented *Clostridium* bins in the metagenome, bins 1 and 4, had uncharacteristic *Clostridium fhs* genes that were instead assigned to other clostridial genera. So, the *fhs* of *Clostridium* bin 1 showed a high amino acid sequence similarity to that of *Clostridium* sp. WB02 MRS01, which does not fall under *Clostridium* genus *sensu stricto* and is known as *Lacrimispora celerecrescens* (>99.2% amino acid identity), whereas the *fhs* of *Clostridium* bin 4 was far related to *Sedimentibacter saalensis* (80.5% amino acid identity).

**FIGURE 7:**
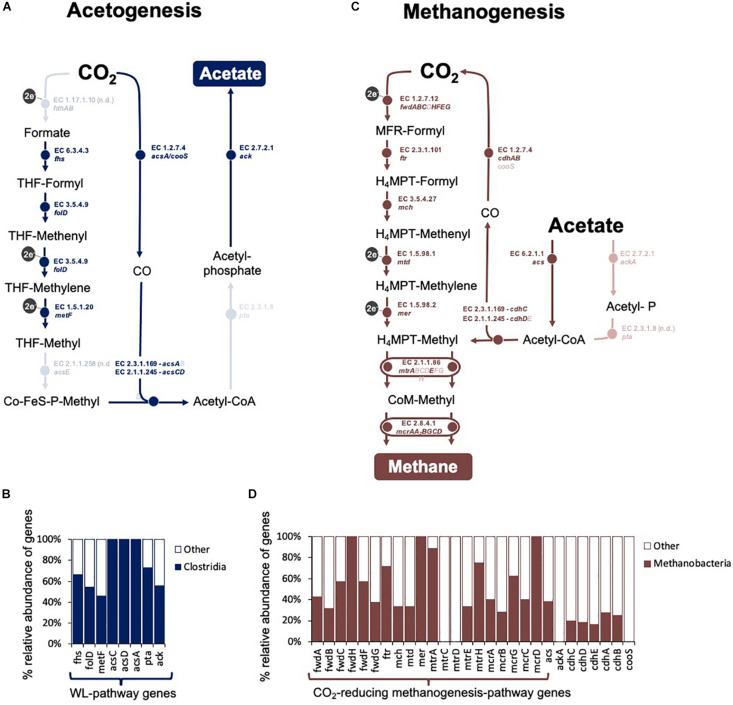
Overview of CO_2_-reducing acetogenesis and methanogenesis in an assembled metagenome obtained at the fourth transfer of the Fe^0^-corrosive enrichment. Only reactions involved in C-modification during **(A,C)** acetogenesis and **(B,D)** methanogenesis are presented. **(A)** The Wood Ljungdahl pathway in the *Clostridial* metagenome. It includes: *fdh* operon (formate dehydrogenase), *fhs* (formate–tetrahydrofolate ligase), *folD* (methenyltetrahydrofolate cyclohydrolase), *metF* (methylenetetrahydrofolate reductase), *acsE* (5-methyltetrahydrofolate:corrinoid/iron-sulfur protein Co-methyltransferase), *acs* (CO-methylating acetyl-CoA synthase), *acsCD* (5-methyltetrahydrosarcinapterin:corrinoid/iron-sulfur protein CO-methyltransferase), *acsA/cooS* (Ni CO dehydrogenase), pta (phosphate acetyltransferase), and ack (acetate kinase). **(B)** Relative distribution of the genes for the WL-pathway in Clostridia. **(C)** Methanogenesis pathway in the *Methanobacteria* metagenome. It includes: *fmd/fwd* operon (formylmethanofuran dehydrogenase), *ftr* (formate–tetrahydrofolate ligase), *mch* (N^5^, N^10^-methenyltetrahydromethanopterin cyclohydrolase), *mtd* (F_420_-dependent N^5^, N^10^-methylene-H4MPT dehydrogenase), *mer* (N^5^, N^10^-methylene-H_4_MPT reductase), *mtr* operon (coenzyme M methyltransferase), *mcr* operon (methyl:coenzyme M reductase), *hdrA_1_B_1_C_1_* (ferredoxin:CoB—CoM heterosulfide reductase), *mvhAGD* (*-[NiFe]-hydrogenase*, *eha* operon (energy-converting hydrogenase A), *ehb* operon (energy-converting hydrogenase B), and *frhABG* (F_420_-reducing hydrogenase) for CO_2_-reducing methanogenesis, plus *cdhED* (acetyl-CoA decarbonylase), *cdhC* (CO-methylating acetyl-CoA synthase), *acs* (acetyl-CoA synthetase), *ackA* (acetate kinase), and *pta* (phosphotransacetylase) for accetotrophic methanogenesis. **(D)** Relative distribution of the genes for the CO_2_-reducing methanogenesis pathway and acetoclastic methanogenesis pathways in *Methanobacteria*. Lighter shades show steps that had no hits for either *Clostridia* or *Methanobacteria*.

Overall, class *Clostridia* showed the best representation of the Wood Ljungdahl pathway (Figure 7 and Supplementary Table S5).

At the 11th transfer on Fe^0^, the lake community became entirely governed by bacteria of the class *Clostridia* according to amplicon sequencing of the V4 region of the 16S rRNA gene. We could not detect any *Proteobacteria* and *Bacteroidia/Parabacteroidia* during this latter transfer, indicating that only *Clostridia* species survived. We could not match the V4 region to previously identified bins because neither included a complete 16S rRNA gene.

*Clostridia* of the genus *Clostridium* were best represented in this enrichment’s metagenome after four transfers (36%, Supplementary Figure S5) and persisted in the enrichments after 11 transfers. *Clostridium* includes several species of CO_2_-reducing acetogens that use H_2_ as an electron donor (Bengelsdorf et al., 2018) but also electrodes (Nevin et al., 2011) and Fe^0^ (Monroy et al., 2011). Besides, *Clostridium* was previously enriched on Fe^0^ from environments such as scraped bicycles thrown in a Dutch channel (Philips et al., 2019), rice paddies (Kato et al., 2015), or oilfield production waters (Ma et al., 2019). These studies, along with ours, suggest that *Clostridium* is a likely corrosive acetogen when Fe^0^ becomes available in their environment.

*Clostridia* of the genus *Acetobacterium* were not represented in the metagenome after four transfers but were the main amplicon OTU detected after 11 transfers (Figure 5 and Supplementary Table S5). Nevertheless, whether this organism is a true *Acetobacterium* or a *Clostridium* remains to be determined. Phylogenetic assignment by short 16S amplicon sequences, although effective for most organisms, is ineffective for phylogenetic assignment of *Clostridium* species, which fall out of their family when a 16S classification is used (Wiegel et al., 2006) and require up to 46 marker genes for proper phylogenetic classification, which does not include the 16S rRNA gene (Yu et al., 2019).

Still, *Acetobacterium* is another genus within *Clostridia* that includes at least nine species of CO_2_-reducing acetogens (Bengelsdorf et al., 2018). A new species can even utilize Fe^0^ as a sole electron donor (Philips et al., 2019). Still, generally *Acetobacterium* species have not been effective at using Fe^0^ (Kato et al., 2015) or electrodes (Nevin et al., 2011) as electron donors. Yet, *Acetobacterium* species are often enriched on cathodes (Marshall et al., 2012, 2013; Labelle et al., 2014; Patil et al., 2015) and sometimes on steel (Mand et al., 2014; Philips et al., 2019). Moreover, they have been found in corrosive biofilms on steel structures from oil production facilities (Vigneron et al., 2016). It is believed that they were non-corrosive and rather feeding syntrophically. Thus, the exact role of *Acetobacterium* in Fe^0^ corrosion remains to be determined.

Essentially, the class *Clostridia* includes the majority of the CO_2_-reducing acetogens (50 out of 61 described CO_2_-reducing acetogens) (Bengelsdorf et al., 2018), some of which are known Fe^0^ corroders and were also the main contenders at acetogenesis in our Fe^0^-dependent enrichments according to the distribution of the key gene for acetogenesis (Supplementary Figure S5).

#### Methanogens

When the corrosive community reached the fourth transfer on Fe^0^, using shotgun metagenomics, we identified two major methanogenic groups: *Methanomicrobia* (48% of Archaea; 3.2% of assembled prokaryotic reads, 1 assembled bin) and *Methanobacteria* (38.5% of Archaea; 2.6% of assembled prokaryotic reads, 1 assembled bin). The *Methanobacteria* metagenome reads provided the best coverage of the CO_2_-reducing methanogenesis pathway (Supplementary Table S6 and Figure 7).

We could assemble two bins or metagenome-assembled genomes, one *Methanomicrobia* related to *Methanosaeta/Methanothrix*, and the other a *Methanobacteria* related to *Methanobacterium/Methanothermobacter*. MG-RAST assigned the *Methanobacteria* bin to *Methanothermobacter*, a former *Methanobacterium* (Wasserfallen et al., 2000). However, analyses of the key gene for methanogenesis (*mcrA*—for methyl coenzyme M reductase) designated the bin to a *Methanobacterium* (Figure 6 and Supplementary Table S3). In fact, the two *mcrA*s in the *Methanobacteria-*bin 9 were more similar to the *mcrA* of described *Methanobacterium* species, one being 98% identical to the *mcrA* of *M. formicicum*, the other 96.6% to *Methanobacterium* sp. NBRC 105039. Both were only distantly related to the *mcrA* of *Methanothermobacter thermoautotrophicus* with 89.3 and 85.5% amino acid identity, respectively. The *mcrA* of *Methanosaeta/Methanothrix* bin eight was very closely related to that of *Methanothrix shoehngenii* (99.5% amino acid identity).

At the 11th transfer on Fe^0^, the lake community was represented solely by *Methanobacterium* according to amplicon sequencing of both the V4 and V3–V5 regions of the 16S rRNA gene. We could not detect any *Methanomicrobia* during this latter transfer, indicating that only *Methanobacterium* species survived 11 transfers on Fe^0^. Unfortunately, we could not match the V4 or V3–V5 regions to previously identified *Methanobacterium* bins because they did not contain a 16S rRNA gene.

*Methanobacterium/Methanothermobacter* are often associated with biofilms formed on Fe^0^ infrastructure like corroded sea-submerged railway lines (Usher et al., 2014b) or oil pipelines and oil facility infrastructure (Duncan et al., 2009; Lenchi et al., 2013; Liang et al., 2014; Okoro et al., 2017; Suarez et al., 2019; Lahme et al., 2020; Su et al., 2020). Sometimes, *Methanobacterium/Methanothermobacter* are implicated in severe corrosion (Lahme et al., 2020). Other times are thought to help form a protective layer against corrosion (in’t Zandt et al., 2019). Several corrosive *Methanobacterium* have been isolated from various environments, like North sea coastal sediments (Dinh et al., 2004) or an oil storage tank in Japan (Uchiyama et al., 2010), and several *Methanothermobacter* species (formerly known as thermophilic *Methanobacterium*) have been previously shown capable of Fe^0^ corrosion in pure culture [e.g., *M. thermoautotrophicum* (Daniels et al., 1987; Karr, 2013) or *M. thermoflexus* (Mayhew et al., 2016)]. On the other hand, a *Methanothermobacter* strain isolated from a corroded oil pipeline could not corrode Fe^0^ alone and required partnering with other microorganisms (Davidova et al., 2012).

Recently, Dutch researchers suggested that *Methanobacteriales*, including *Methanobacterium* and *Methanothermobacter*, have anticorrosive properties (in’t Zandt et al., 2019). It has been afterward debated whether *Methanobacterium/Methanothermobacter* induce Fe^0^ corrosion. However, in our enrichments, methanogens, best represented by *Methanobacterium*/*Methanothermobacter*, have an active role in Fe^0^ corrosion within the microbial community, upholding Fe^0^-dependent methanogenesis months after acetogenic activity ceased (Figure 3).

### Possible Mechanism of Electron Uptake During Fe^0^ Corrosion

Methanogens showed four to five times higher rates in the presence of acetogens than alone, when methanogenesis rates were fully explained by abiotic H_2_. It is therefore unlikely that methanogens in this corrosive microbiome have an inherent mechanism of electron uptake from Fe^0^. Indeed, we found no evidence in the metagenome that methanogens had the potential to accelerate electron uptake from Fe^0^ as they did not present the MIC island specific to highly corrosive methanogens (Tsurumaru et al., 2018). [NiFe]-hydrogenases on the MIC island of methanogens promotes corrosion only when encoded on the genomic island (MIC island) between secretory proteins, which apparently help the hydrogenase on its way out of the cell to the extracellular electron donor (Tsurumaru et al., 2018). Hydrogenotrophic methanogens also contain an enzyme supercomplex (Mvh/Hdr: methyl viologen reducing hydrogenase/heterodisulfide reductase) required for energy metabolism, which can function outside the cells evolving H_2_ or formate from Fe^0^ (Lienemann et al., 2018). Nonetheless, there is no supporting evidence that the Hdr supercomplex gets excreted or that it has a particular role in corrosion by methanogens since all hydrogenotrophic methanogens contain this supercomplex, but not all are corrosive.

Methanogens in this corrosive microbiome appear to benefit from the presence of acetogens by mechanisms that could not be fully explained by acetate turnover.

To further explain the methanogens’ dependency on acetogens, we looked into possible strategies acetogens may use to enhance extracellular electron retrieval from Fe^0^, which would also benefit neighboring cells. These are:

(1) enzymes (e.g., hydrogenases) lowering the activation energy for Fe^0^ electron oxidation to form H_2_ (Deutzmann et al., 2015; Rouvre and Basseguy, 2016; Lienemann et al., 2018).
(2) cell-associated metals and peptides may act as organometallic catalysts lowering the activation energy for the H_2_ evolution reaction. In fact, streamlined organometallic molecules (e.g., diiron oxadithiolate) mimicked the chemistry of the H_2_ evolution reaction by hydrogenase enzymes (Song et al., 2005). Interestingly, biocathodes with killed cell biomass retained peptides and metals on the cathode surface promoting H_2_ evolution (Yates et al., 2014). These previous observations together mean that organometallic-rich cell deposits on the Fe^0^ surface may act similar to hydrogenases in promoting the H_2_ evolution reaction.
(3) shuttles (e.g., flavins) may cycle electrons between Fe^0^ and cells and induce corrosion. Shuttles (e.g., flavins) have been involved in extracellular electron transfer in many Firmicutes including *Clostridia* (Light et al., 2018), but their role in corrosion and whether they could be used by methanogens have, to our knowledge, never been reported.

In our enrichments, cell filtrate from a 10-day-old culture stimulated four-fold the H_2_ evolution reaction from Fe^0^ and three-fold when heat inactivated (Figure 4). Thus, the spent media filtrate contains excreted thermolabile and non-thermolabile catalysts that promote the H_2_ evolution reaction. The thermolabile cell exudates could be enzymes or shuttles (e.g., flavins) while the non-thermolabile constituents could be metal/peptide cell debris.

Because thermolabile constituents from the media (e.g., hydrogenases, nitrogenases) partially explained enhanced H_2_ evolution from Fe^0^, we screened the assembled metagenome for enzymes and shuttles.

**FIGURE 8:**
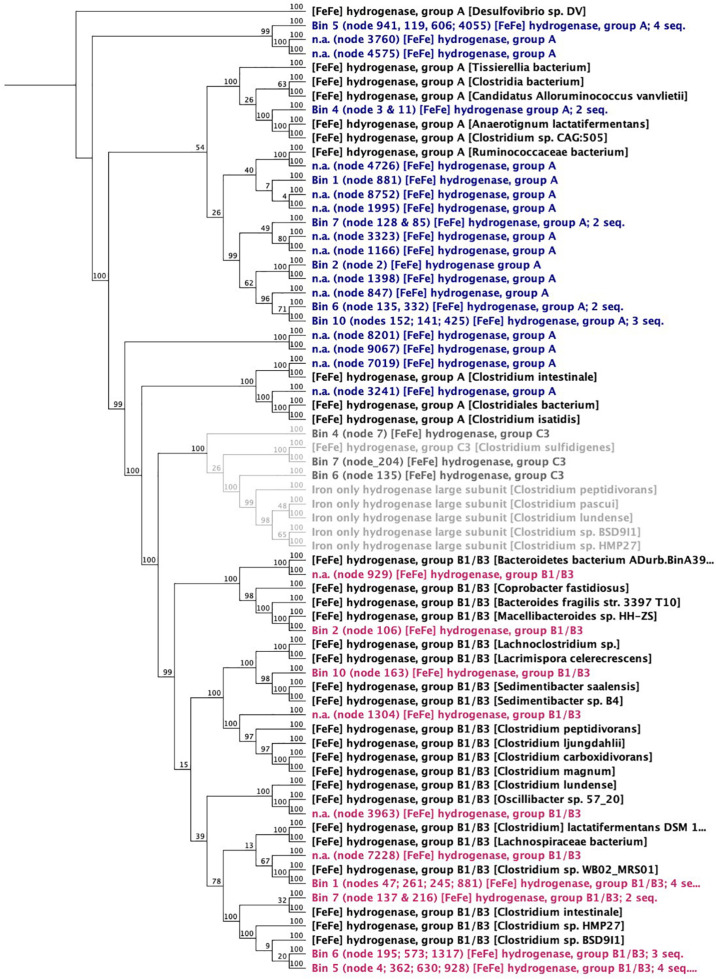
Neighbor joining dendrogram of amino acid sequences of [FeFe]-hydrogenases. Numbers close to nodes indicate bootstrap probability (%) based on 100 replicates. The outgroup root is a *Deltaproteobacteria*—a *Desulfovibrio* sp.

**Enzymes:** In biological systems, H_2_-evolving reactions are typically catalyzed by ferredoxin-dependent hydrogenases (EC: 1.12.7.2 like the diiron-containing *Clostridial* hydrogenases ([FeFe]-hydrogenases). The [FeFe]-hydrogenases of acetogens have characteristic high H_2_ evolution rates (Adams, 1990) and take up electrons directly from Fe^0^ (Mehanna et al., 2008; Rouvre and Basseguy, 2016). Other H_2_-evolving enzymes are nitrogenases, which release H_2_ as a side product of the dinitrogen fixing reaction and have been confirmed by genome-wide transcriptomics to play a role in H_2_ evolution in *Clostridium* species (Calusinska et al., 2015). Both [FeFe]-hydrogenase and nitrogenases were well represented in the *Clostridia* in this corrosive microbiome (Figure 8 and Supplementary Figure S6). In fact, [FeFe]-hydrogenase enzymes (classes A–C) could be matched to all six *Clostridium* bins and also to one *Parabacteroides* bin (Figure 8).

One possible candidate for effective H_2_ evolution from Fe^0^ is the ferredoxin-dependent hydrogenases (hydrogen-evolving hydrogenase HydA; EC:1.12.7.2). When screening the metagenome, HydA was found exclusively (100%) in *Clostridia* (87% affiliated to *Clostridium* and 7% to *Eubacterium*).

Since *Clostridium* excrete enzymes (and toxins) extracellularly to carry out lytic functions (Revitt-Mills et al., 2015), some may have evolved the capacity to excrete hydrogenases, promoting access to insoluble food sources like Fe^0^.

**Shuttles:** Other possible secreted constituents in the spent media are flavins, which can be used as shuttles between cells and Fe^0^. Although reports are scarce (Fuller et al., 2014), *Clostridia* can produce and excrete flavins for extracellular electron transfer. In this corrosive microbiome, we could identify almost a full pathway for the biosynthesis of riboflavin, FMN and FAD in *Clostridia* (Supplementary Figure S7). It is therefore possible that *Clostridia* stimulates electron uptake from Fe^0^ also by using flavin shuttles. However, it is unclear how flavins may stimulate methanogenic rates, since, to our knowledge, there are no reports that flavins act as shuttles to promote methanogenesis.

**FIGURE 9:**
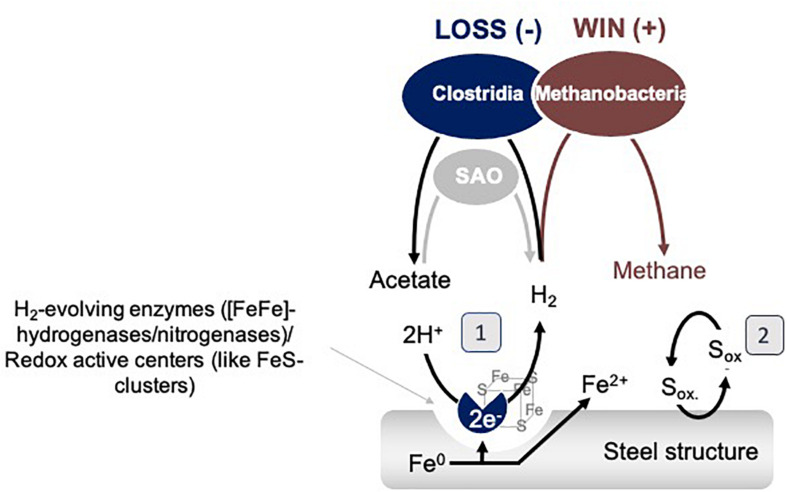
Model interspecies interactions on Fe^0^ between *Clostridia* and *Methanobacteria*. SAO represents syntrophic acetate oxidizing bacteria. (1) represents a mechanism based on catalytic constituents released by cells which could promote the H_2_ evolution reaction, and (2) represents a shuttle based mechanism of interaction.

In other words, the results above could explain why the metabolism of methanogens favorably influenced acetogens. It is most likely that *Methanobacterium* methanogens became stimulated by extracellular *Clostridial* enzymes, which promote, without discrimination, any H_2_/CO_2_ metabolisms in the enrichment. Once outside the cell, extracellular *Clostridial* enzymes capture Fe^0^ electrons to evolve H_2_. H_2_ is then a common resource. Thus, excreted and freely available catalytic molecules that promote the H_2_ evolution reaction on Fe^0^ support a methanogen/win–acetogen/loss scenario (Figure 9).

Possible explanations for why *Methanobacterium* become even more favored by the termination of acetogenesis are that: (1) during the late stationary, *Clostridial* cells lyse, releasing more enzymes/redox-active proteins and thereby promoting H_2_ evolution and the growth of hydrogenotrophic methanogens, or (2) some *Clostridia* switch to syntrophic acetate oxidation (SAO) engaging *Methanobacterium* further as a syntrophic partner (Figure 9).

Additional examinations of the interactions at the Fe^0^ surface and the identity of the thermolabile and non-thermolabile constituents that promote H_2_ evolution from Fe^0^ remain to be resolved in future studies.

The bottle experiments presented here mimic the static freshwater environment of lake sediment conditions, informing about the type of microorganisms and interactions that may accelerate corrosion in such environments. However, we cannot juxtapose these bottle results to a natural environment, where other environmental factors may play a role. Therefore, *in situ* studies of corrosion in such environments are necessary and could be guided by a better understanding of the corrosive species present in these environments.

## Conclusion

Interspecies interactions on Fe^0^ have been insufficiently investigated. Here, we bring evidence for a win–loss type of interaction on Fe^0^ between methanogens (esp. *Methanobacterium*) and acetogens (*Clostridia*) enriched from climate lake sediments.

Acetogens were more effective Fe^0^ corroders and acetate producers when decoupled from methanogens with the help of a specific inhibitor. Methanogens, on the other hand, became less effective methane producers when decoupled from acetogens with a specific inhibitor.

When both groups were together, the intact community exhibited metabolic rates beyond what abiotic H_2_ could explain. Since *Clostridia* are known to release molecules and enzymes extracellularly, we tested whether extracellular enzymes/shuttles may mediate H_2_ evolution from Fe^0^. Undeniably, filtered spent media filtrate promoted H_2_ evolution from Fe^0^ four-fold compared to abiotic controls, partially via thermolabile constituents (enzymes/flavins), partially via undefined non-thermolabile constituents. We screened the metagenome for potential enzyme candidates and identified *Clostridial* [FeFe] hydrogenases, known to be effective in retrieving electrons directly from Fe^0^. Additionally, we found an almost complete flavin-biosynthesis pathway in *Clostridia*. We could not find genomic evidence for enzymatic mediated electron uptake typical of certain methanogens (the MIC island was absent). Besides, it was puzzling that methanogenic rates jumpstarted (above what is expected from abiotic H_2_) only when methanogens co-existed with acetogens, especially when acetogens ceased their activity. The higher rates could be somewhat explained by the late stationary release of extracellular *Clostridial* hydrogenases that promote H_2_ evolution. Another explanation is the metabolic reversal of some *Clostridial* metabolism from CO_2_-reducing acetogenesis to acetate oxidation when the *Clostridia* would have to be coupled syntrophically to the methanogens. These suggestions need to be further validated experimentally.

Some studies suggested that methanogens like *Methanobacteria* may have a protective role during corrosion (in’t Zandt et al., 2019). Here, we show that such methanogens can upkeep corrosion in biofilms where acetogens are present, even after acetogenesis ceases. Therefore, we recommend that the corrosive effects of methanogens should be investigated not only in pure culture but also in consortia with acetogenic partners before suggesting they have a protective anticorrosive role.

## Data Availability Statement

The datasets presented in this study can be found in online repositories. The names of the repository/repositories and accession number(s) can be found in the article/Supplementary Material.

## Author Contributions

A-ER and PP developed the idea. PP carried out all wet-lab experimental work and some of the analyses and drafted the manuscript. A-ER carried out most of the data analyses and wrote the manuscript. WF carried out the metagenome assembly and some of the analyses. All authors contributed to editing the last version of the manuscript.

## Conflict of Interest

The authors declare that the research was conducted in the absence of any commercial or financial relationships that could be construed as a potential conflict of interest.
