# Supplementary material for: A Win–Loss Interaction on Fe0 Between Methanogens and Acetogens From a Climate Lake
Source: Front Microbiol. 2021 May 13;12:638282. doi: 10.3389/fmicb.2021.638282 (PMC8158942; doi:10.3389/fmicb.2021.638282)
Supplement: Supplementary file 1 [file Data_Sheet_1.PDF]

Supplementary material

A win-loss interaction on Fe^0 between methanogens and acetogens from a climate lake

Paola Andrea Palacios [1], Warren R. Francis[1] and Amelia-Elena Rotaru[1*]

[1]Nordcee, Department of Biology, University of Southern Denmark, Odense, Denmark

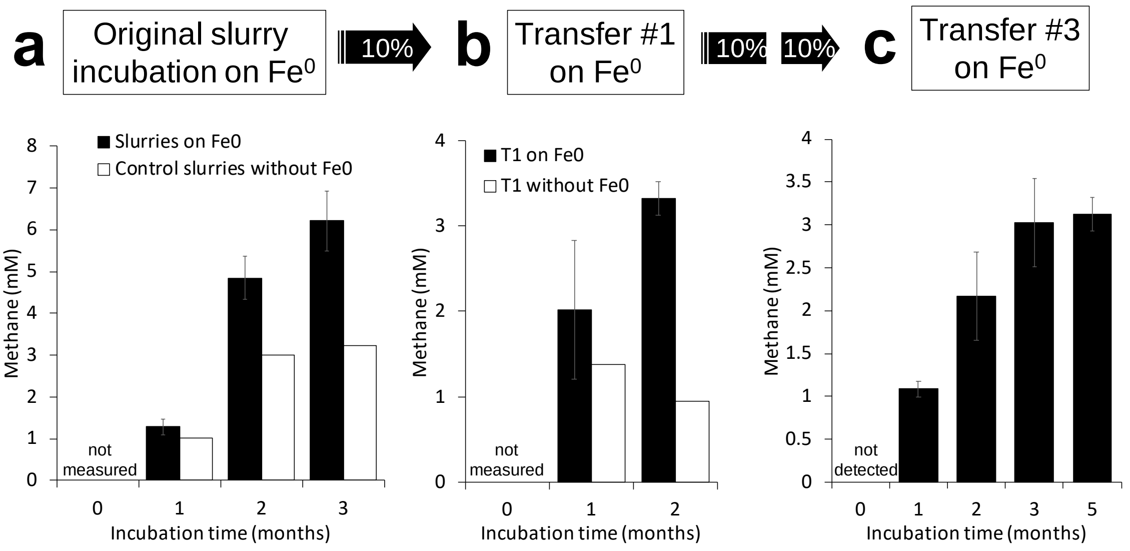

Figure 1S Methane production during transfers carried over > 1 year of incubations on Fe^0. a) Methane production by the original slurries (n=3) initiated with lake sediment from the 15-20 cm horizon and Fe^0 as additional electron donor, versus a slurry without Fe^0 from the same sediment (n=1); b) the first transfer of triplicate enrichments (n=3) with a 10% slurry inoculum and Fe^0 as the only electron donor; the control incubation was carried out without Fe^0 verifying for the presence of any carryover substrates; c) the third transfer on freshwater Fe^0 media of triplicate enrichments with Fe^0 as the only electron donor.

Table 1S. Unassembled shotgun metagenome statistics

	# Seq.	%
**Sequences total**	4,032,354	100.00%
**Sequences that passed QC**	3,723,388	90.58%
**Sequences that failed QC**	308,966	7.66%

Sequences that passed QC	# Seq.	%	Post QC seq. length
**Post QC: sequences total**	3,723,388	100.00%	250 ± 35 bp
**Post QC: rRNA genes**	35,738	1.00%	
**Post QC: proteins with known function**	3,337,130	90.58%	
**Post QC: proteins with unknown function**	311,188	8.45%	

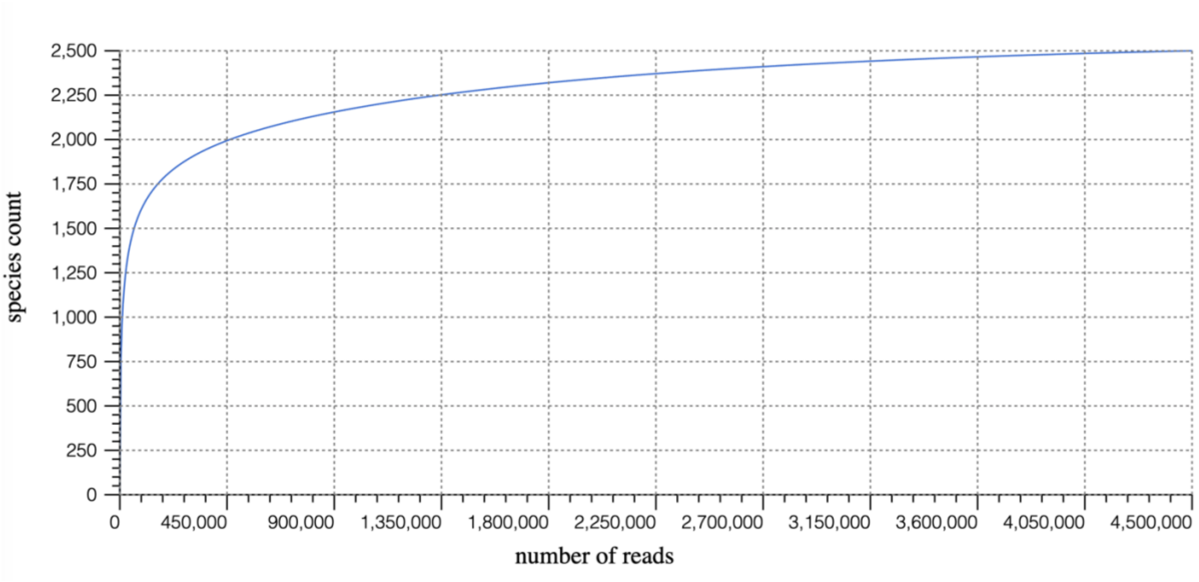

Figure 2S. A rarefaction curve of the annotated species as a function of the number of sequences sampled. Since the curve became flattened to the right a significant number of sequences were sampled and additional sampling is unlikely to yield additional species.

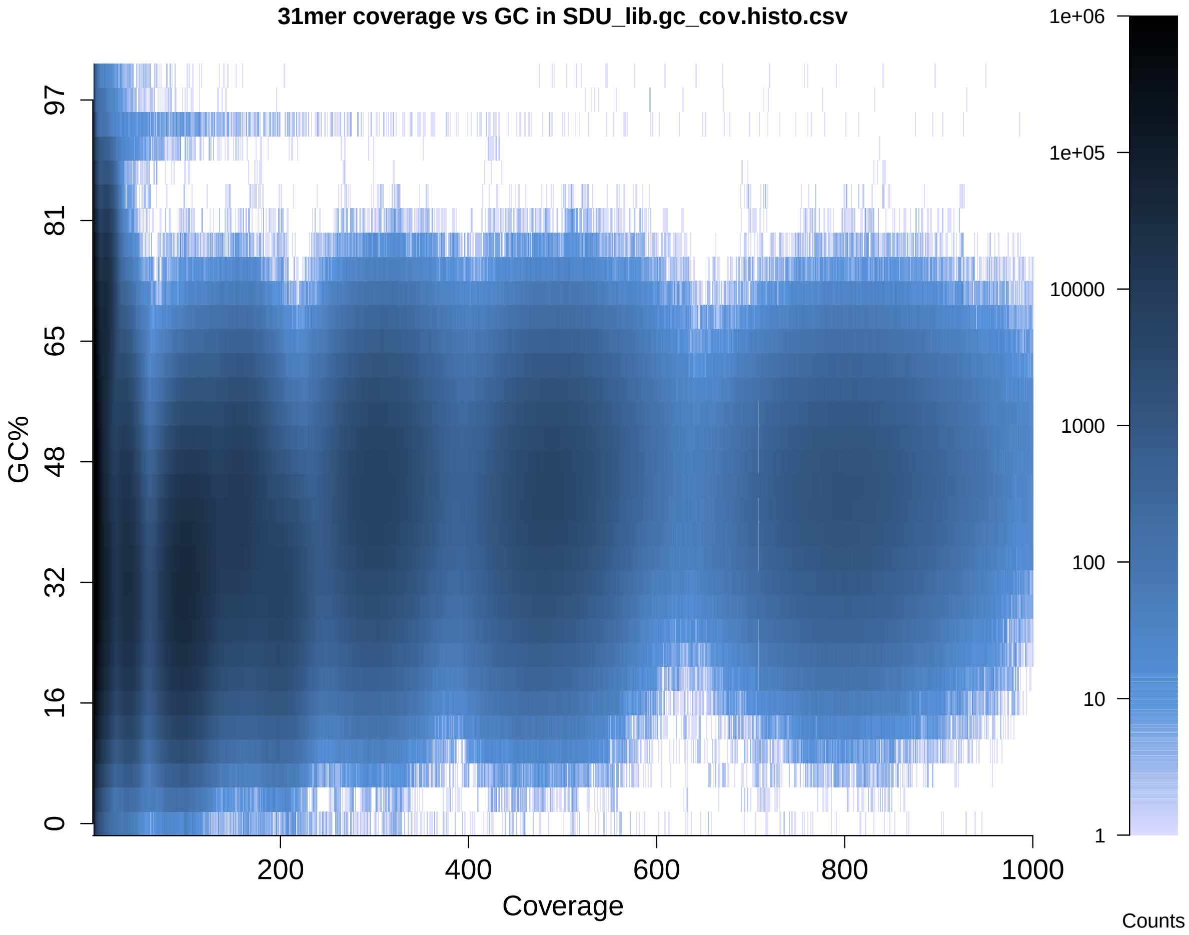

Figure 3S. Coverage of 31mers. Higher counts are darker color.

Table 2S. Metagenome assembled genomes of acetogenic and methanogenic microorganisms from climate lake enrichments transferred four times only with Fe^0 as electron donor.

Bin #	Taxonomic assignment[a]	Contigs	Total Mb	Species	Mean coverage[c]	Genes	Genes w. KEGG annotation	N_{50} (kb)
1	*Clostridium*	365	9.39	2	541.07	8776	4894	61.3
2	*Bacteroides/Parabacteroides*	67	4.17	1	125.61	3396	1618	97.9
3	*Desulfovibrio*	145	3.1	1	11.47	2917	1565	63.2
4	*Clostridium*	30	2.66	1	92.89	2584	1411	278.9
5	*Clostridium*	603	10.97	2	115.68	10600	5020	43.5
6	*Clostridium*	105	2.96	1	45.79	2909	1492	62.0
7	*Clostridium*	265	3.9	1	11.55	3915	1771	45.2
8	*Methanosaeta*	115	2.26	1	4.87	2328	1155	32.9
9	*Methanothermobacter/Methanobacterium*[b]	40	1.35	1	5.9	1364	658	63.9
10	*Clostridium*	460	5.12	1	5.84	5197	2318	60.5

[a]Taxonomic assignment is based on MG-RAST annotation. In parenthesis we added reassignment after analyses of key genes for acetogenesis and methanogenesis against GenBank protein database.

[b]MAG assembled by hit filtering.

[c]The coverage was calculated as part of the SPAdes assembler, the mean coverage was calculated as Σ(contig average * contig length)/ Σ(contig length). Detailed information is available on github: https://bitbucket.org/wrf/corrosion-community-2021/src/master/blobplot_metagenome_gc_cov_binned.R

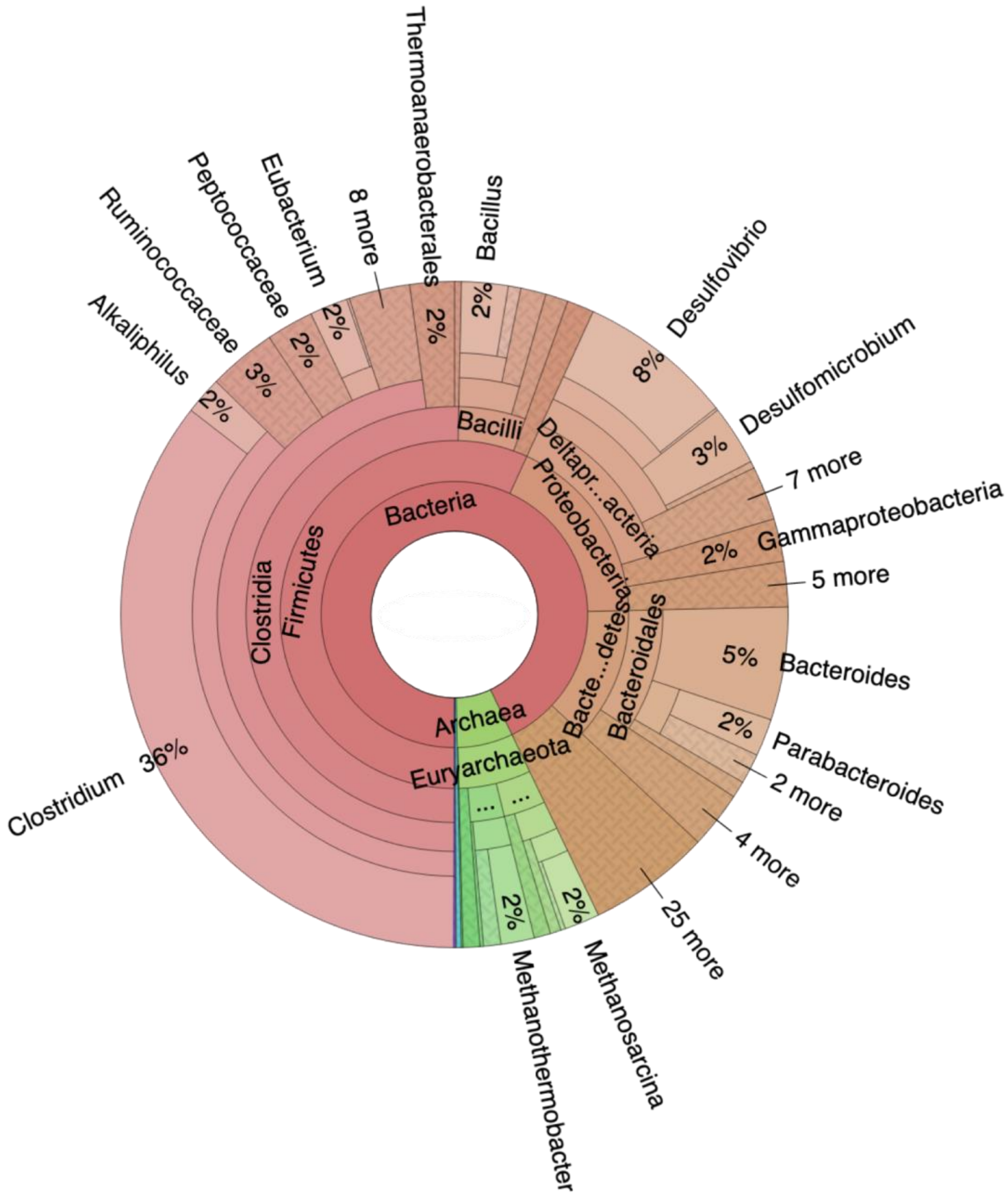

Figure 4S. Krona plot of taxa distribution at the genus level against the NCBIs RefSeq database; Parameters used: e-value 10^{-5}, minimum percent-identity 80%; minimal alignment length 15bp.

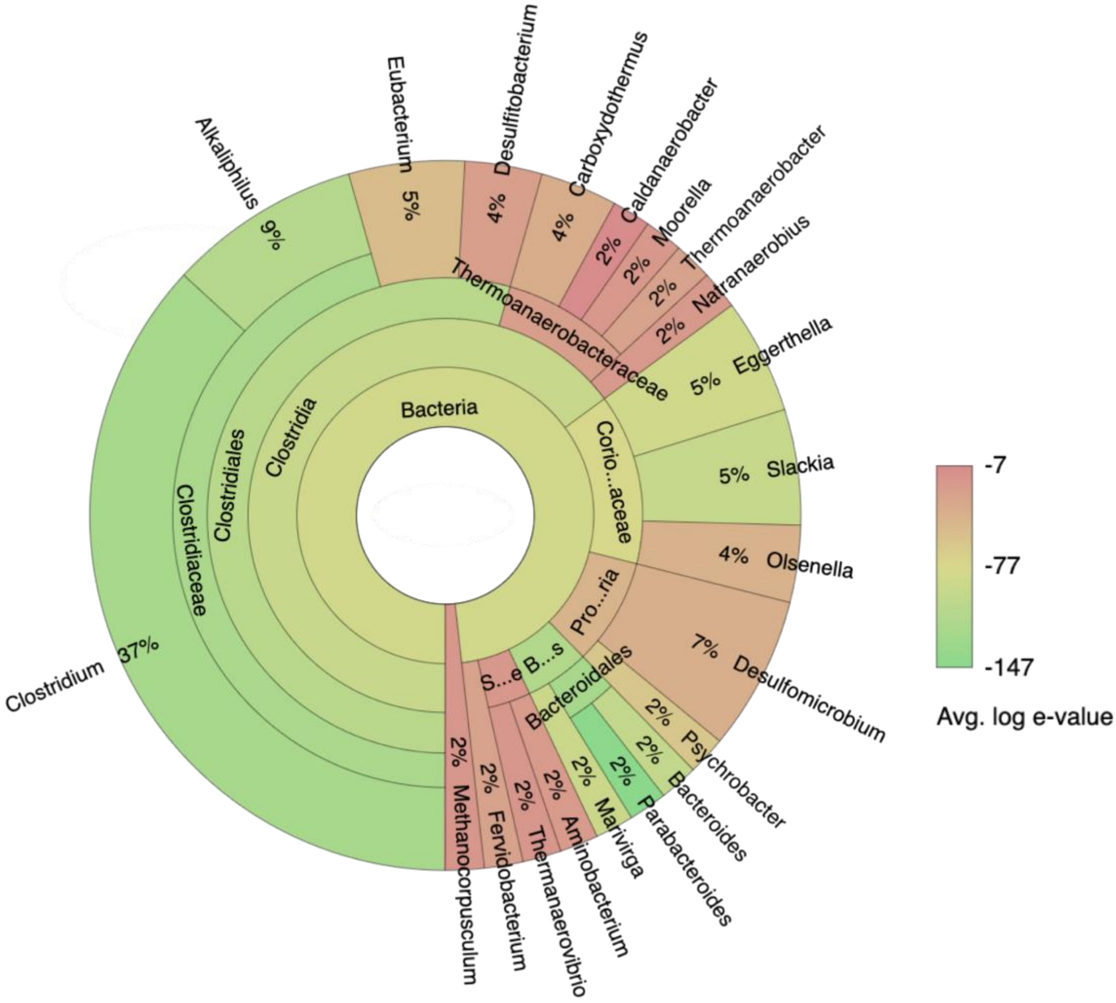

Figure 5S. Krona plots of distribution among taxa of the *fhs* formyltetrahydrofolate synthetase (EC 6.3.4.3), the key enzyme in the acetyl-CoA pathway of acetogenesis.

Table 3S. Aminoacid identity for the key gene for acetogenesis, Fhs (formyltetrahydrofolate synthetase) and the key gene for methanogenesis, McrA (methyl-coenzyme M reductase, alpha subunit, also known as coenzyme-B sulfoethylthiotransferase, alpha subunit).

MG-RAST annotation	Bin	Node	Gene hit	Closest relative	max score	total score	coverage	e-value	% identity	lenght	Accession
Clostridium	bin 1	node_49	formate--tetrahydrofolate ligase [Clostridium sp. WB02_MRS01]	Clostridium sp. WB02_MRS01	1125	1125	100%	0	99.28%	556	WP_154520701.1
Clostridium	bin 1	node_1485	formate--tetrahydrofolate ligase [Clostridium sp. WB02_MRS01]	Clostridium sp. WB02_MRS01	928	928	100%	0	99.56%	556	WP_154520701.1
Bacteroides/Parabacteroides	bin 2	node_2	formate--tetrahydrofolate ligase [Macellibacteroides sp. HH-ZS]	Macellibacteroides sp. HH-ZS	1051	1051	100%	0	93.15%	555	OCW92970.1
Clostridium	bin 4	node_12	TPA: formate--tetrahydrofolate ligase [Lachnospiraceae bacterium]	Sedimentibacter saalensis	924	924	100%	0	80.54%	560	WP_145085905.1
Clostridium	bin 5	node_794	formate--tetrahydrofolate ligase [Clostridium tunisiense]	Clostridium tunisiense	1103	1103	100%	0	98.02%	556	WP_017414754.1
Clostridium	bin 5	node_984	formate--tetrahydrofolate ligase [Clostridium lundense]	Clostridium lundense	1129	1129	100%	0	99.46%	560	MBE6069199.1
Clostridium	bin 7	node_637	formate--tetrahydrofolate ligase [Clostridium sulfidigenes]	Clostridium sulfidigenes	1123	1123	100%	0	99.82%	556	MBE6059686.1
Clostridium	bin 7	node_9395	formate--tetrahydrofolate ligase [Sedimentibacter hydroxybenzoicus]	Sedimentibacter hydroxybenzoicus	941	941	100%	0	90.56%	561	WP_179238276.1
Methanosaeta	bin 8	node_565	coenzyme-B sulfoethylthiotransferase subunit alpha [Methanothrix so	Methanothrix soehngenii	1162	1162	100%	0	99.46%	559	WP_013718622.1
Methanobacterium	bin 9	node_80	coenzyme-B sulfoethylthiotransferase subunit alpha [Methanobacteriu	Methanobacterium formicicum	1130	1130	100%	0	98.00%	550	WP_048072491.1
Methanobacterium	bin 9	node_80	coenzyme-B sulfoethylthiotransferase subunit alpha [Methanothermo	Methanothermobacter thermautotrophicus	1023	1023	100%	0	89.27%	550	WP_192962010.1
Methanobacterium	bin 9	node_42	methyl-coenzyme M reductase, alpha subunit [Methanobacterium sp.	Methanobacterium sp. Maddingley MBC34	1129	1129	100%	0	98.18%	550	EKQ54655.1
Methanobacterium	bin 9	node_42	coenzyme-B sulfoethylthiotransferase subunit alpha [Methanothermo	Methanothermobacter thermautotrophicus	984	984	99%	0	85.45%	553	WP_192962033.1

Table 4S. Relative amplicon abundance at the 11th transfer on Fe^0 and the closest culture relatives of the OTUs.

	Amplicon relative abundance												
Total	Culture 1	Culture 2	Culture 3	OTU	Affiliation	Relative in culture	Max score	Total score	Querry cover	e-value	% identity	Accession	Other characteristics
OTU_1	17363	17408	21768	OTU_1	(Methanobacteria) Methanobacterium #1	Methanobacterium subterraneum	514	1028	100%	5.00E-143	98.29%	CP017768.1	methanogen from subseafloor
OTU_5	1795	2646	3380	OTU_5	(Clostridia) Acetobacterium	Acetobacterium carbinolicum subsp. kysingense	518	518	100%	4.00E-144	98.63%	NR_115316.1	acetogen
OTU_10	1638	1221	1373	OTU_10	(Methanobacteria) Methanobacterium #2	Methanobacterium palustre	514	514	100%	5.00E-143	98.29%	NR_041713.1	methanogen from ricefield
OTU_25	1127	975	1142	OTU_25	(Methanobacteria) Methanobacterium #3	Methanobacterium ferruginis	525	525	100%	2.00E-146	98.98%	NR_113045.1	corrosive methanogen from oil facilities
OTU_85	790	738	1087	OTU_85	(Methanobacteria) Methanobacterium #4	Methanobacterium subterraneum	520	1040	100%	1.00E-144	98.63%	CP017768.1	methanogen from subseafloor
OTU_12	356	723	713	OTU_12	(Clostridia) Sedimentibacter	Sedimentibacter saalensis	449	449	100%	1.00E-123	94.52%	NR_025498.1	reclassified Clostridium
OTU_14	225	419	741	OTU_14	(Clostridia) Clostridium #1	[Clostridium] sphenoides JCM 1415	518	3103	100%	4.00E-144	98.63%	LT630003.1	
OTU_32	58	151	210	OTU_32	(Clostridia) Clostridium #2	[Clostridium] Anaerotignum propionicum DSM 1682	462	3239	100%	2.00E-127	95.22%	CP014223.1	
OTU_171	36	32	36	OTU_171	(Methanobacteria) Methanobacterium #5	Methanobacterium subterraneum	459	918	92%	3.00E-126	97.06%	CP017768.1	methanogen from subseafloor
OTU_163	29	51	40	OTU_163	(Clostridia) Clostridium #3	Clostridium tepidum	429	429	100%	2.00E-117	93.17%	NR_157639.1	
OTU_42	0	0	0	OTU_42	(Clostridia) Clostridium #4	Clostridium magnum DSM 2767	501	501	100%	4.00E-139	97.61%	NR_119084.1	

Table 5S. Gene coverage of the Wood Ljungdahl pathway in the assembled metagenome from a lake community transferred 4 times on Fe^0. In bold are the taxons assembled into bins.

Class/<br>Genus<br>(fold bin coverage)	Gene coverage of the Wood Ljungdahl pathway[a]<br>(% relative abundance T4)	Described lithoautotrophic acetogens
***Clostridia/*<br>*Clostridium*[b,c]<br>(6 bins, 5.8 x to 541 x MAG coverage)**	**8 genes**:<br>*fhs* (37.5%); *folD* (40%); *metF* (14.3%); *acsA* (47.8%); *cooS* (69.4%); *acsC* (33.3%); *acsD* (83.3%); *ackA* (36.6%)	12 species (*C. aceticum, C. autoethanogenum, C. carboxidivorans, C. coskatii, C. difficile, C. drakei, C. formicoaceticum, C. ljungdahlii, C. magnum, C. methoxybenzovorans, C. ragsdalei, C. scatologenes*)
Clostridia/<br>*Eubacterium*	**8 genes**:<br>*fhs* (5.4%); *folD* (2.9%); *metF* (5.7%); *acsA* (30.4%); *cooS* (6.5%); *acsC* (16.7%); *acsD* (16.7%); *ackA* (2.4%)	2 species (*E. aggregans, E. limosum*)
Clostridia/<br>*Acetobacterium*[d]	**n.d.**	9 species (*A. bakii, A. carbinolicum, A. dehalogenans, A. fimentarium, A. malicum, A. paludosum, A. tundrae, A. wieringae, A. wodii*)
Clostridia/<br>*Moorella*	**2 genes**:<br>*fhs* (1.8%); *folD* (2.9%)	4 species (*M. glycerini, M. mulderi, M. thermoacetica, M. thermoautotrophica*)
Clostridia/<br>*Desulfotomaculum*[b]	**2 genes**:<br>*cooS* (1.6%); *ackA* (2.4%)	1 species (*D. thermobenzoicum*)
Clostridia/<br>*Carboxydothermus*	**1 gene**:<br>*fhs* (3.6%)	3 species (*C. ferrireducens, C. hydrogenoformans, C. pertinax)*
Clostridia/<br>*Thermoanaerobacter*	**1 gene**:<br>*fhs* (1.8%)	1 species (*T. kivui*)

[a]The Wood Ljungdahl pathway includes enzymes encoded by 13 genes which catalyze 10 reactions (see Fig. 7c): *fdhAB* (formate dehydrogenase), *fhs* (formate—tetrahydrofolate ligase; 56 gene hits), *folD* (methenyltetrahydrofolate cyclohydrolase; 35 gene hits), *metF* (methylenetetrahydrofolate reductase; 35 gene hits), *acsE* (5-methyltetrahydrofolate:corrinoid/iron-sulfur protein Co-methyltransferase), *acsAB* (CO-methylating acetyl-CoA synthase; *acsA* – 23 gene hits), *acsCD* (5-

methyltetrahydrosarcinapterin:corrinoid/iron-sulfur protein CO-methyltransferase *acsC* – 6 gene hits; *acsD* – 6 gene hits), *acsA/cooS* (Ni CO dehydrogenase; *cooS* – 62 gene hits), pta (phosphate acetyltransferase), ack (acetate kinase; 41 gene hits).

[b]The 16S rRNA gene was detected in a *Clostridium* and a *Desulfotomaculum*

[c]The relative abundance of *Clostridium* after 11 transfers on Fe^0 was 2.8±1.3% of all prokaryotes (n=3)

[c]The relative abundance of *Acetobacterium* after 11 transfers on Fe^0 was 9.8±1.9% of all prokaryotes (n=3)

Table 6S. Relative abundance of the CO_2-reductive methanogenesis and acetotrophic methanogenesis pathway in the assembled metagenomes after 4 transfers on Fe^0. In bold are the taxons assembled into bins.

**Genus**<br>(fold MAG coverage)	**Gene coverage of**<br>**CO_2 reductive methanogenesis pathway** [a]<br>(% relative abundance)	**Gene coverage of acetotrophic methanogenesis pathway**<br>(% relative abundance)
Methanobacterium [b,c]<br>**(1 bin 5.9 x coverage)**	**17 gene hits**<br>***fwdA* (35.7%), *fwdB* (26.3%), *fwdC* (57.1%), *fwdH* (50%), *fwdF* (14.3%), *fwdG* (25%), *ftr* (50%), *mch* (22.2%), *mer* (50%), *mtrA* (33.3%), *mtrE* (16.7%), *mcrA* (20%), $mcrA_2$ (66.7%), *mcrB* (14.3%), *mcrG* (37.5%), *mcrC* (20%), *mcrD* (71.4%)**	**5 gene hits**<br>*acs* (38.1%), *cdhC* (10%), *cdhD* (18.2%), *cdhA* (27.8%), *cdhB* (25%)
Methanobrevibacter	**10 genes**<br>*fwdH* (50%), *fwdF* (42.9%), *mch* (11.1%), *mtd* (33.3%), *mer* (25%), *mtrA* (44.4%), *mtrE* (16.7%), $mcrA_2$ (11.1%), *mcrG* (12.5%), *mcrC* (20%)	None
Methanosaeta [d]<br>**(1 bin, 4.9 x coverage)**	**10 gene hits**<br>***fwdE* (11.1%), *fwdG* (25%), *ftr* (7.1%), *mch* (11.1%), *mtrC* (100%), *mcrA* (20%), $mcrA_2$ (5.6%), *mcrB* (14.3%), *mcrG* (12.5%), *mcrC* (20%)**	**6 gene hits**<br>*acs* (28.6%), *cdhC* (10%), *cdhD* (9.1%), *cdhE* (8.33%), *cdhA* (11.1%), *cooS* (25%)
Methanosarcina	**10 gene hits**<br>*fwdA* (14.3%), *fwdB* (31.6%), *fwdF* (28.6%), *fwdE* (66.7%), *mch* (22.2%), *mtrA* (11.1%), *mtrD* (33.3%), *mtrE* (33.3%), *mcrB* (57.1%)<br>*mcrG* (12.5%)	**7 gene hits**<br>*ackA* (100%), *cdhC* (50%), *cdhD* (54.6%), *cdhE* (33.3%), *cdhA* (44.4%), *cdhB* (50%)<br>*cooS* (62.5%)

[a]The CO_2-reductive methanogenesis pathway includes 7 C-transforming reactions catalyzed by enzymes /enzyme complexes encoded by 26 genes, while the acetoclastic methanogenesis includes 3 or 4 C-transforming reactions catalyzed by enzymes encoded by ca. 7 genes (see Fig. 7d).

Genes for CO_2-reductive methanogenesis (only for C-transforming reactions): *fmd/fwdABCDHFEG* (formylmethanofuran dehydrogenase, *fwdA*-14, *fwdB*-19, *fwdC*-7, *fwdH*-2, *fwdF*-7, *fwdE*-9, *fwdG*-8 gene hits); *ftr* (formate—tetrahydrofolate ligase, *ftr*-14 gene hits), *mch* (N^5,N^{10}-methenyltetrahydromethanopterin cyclohydrolase, *mch*–9 gene hits), *mtd* (F_{420}-dependent N^5, N^{10}-methylene-H_4MPT dehydrogenase, *mtd*-3 gene hits), *mer* (N^5,N^{10}-methylene-H_4MPT reductase, *mer*-4 gene hits), *mtrABCDEFGH* (coenzyme M methyltransferase, *mtrA*-9, *mtrC*-1, *mtrD*-3, *mtrE*-6 gene hits), $mcrAA_2BGCD$ (methyl:coenzyme M reductase, *mcrA*-5, $mcrA_2$-18, *mcrB*-7, *mcrG*-8, *mcrC*-5, *mcrD*-7);

Genes for acetotrophic methanogenesis: *acs* (acetyl-CoA synthetase, *acs* – 21 gene hits), *ackA* (acetate kinase, *ackA*-3 gene hits), *pta* (phosphotransacetylase), *cdhED* (acetyl-CoA decarbonylase, *cdhD*-11, *cdhE*-12 gene hits), *cdhC* (CO-methylating acetyl-CoA synthase, *cdhC*-10 gene hits), *cdhAB* (acetyl-CoA decarbonylase/synthase, *cdhA*-18, *cdhB*-4 gene hits), *cooS* (carbon monoxide dehydrogenase, *cooS*-8 gene hits)

[b]*Methanobacterium* from the corrosive enrichments were automatically classified by MG-RAST as thermophilic *Methanothermobacter* (formerly classified as *Methanobacterium*), which was challenged by phylogenetic analyses of the key methanogenesis gene, *mcrA*.

[c]The relative abundance of *Methanobacterium* after 11 transfers on Fe^0 was 94.8±1.9% of the full community (n=3)

[d]The 16S rRNA gene was only detected in *Methanosaeta.*

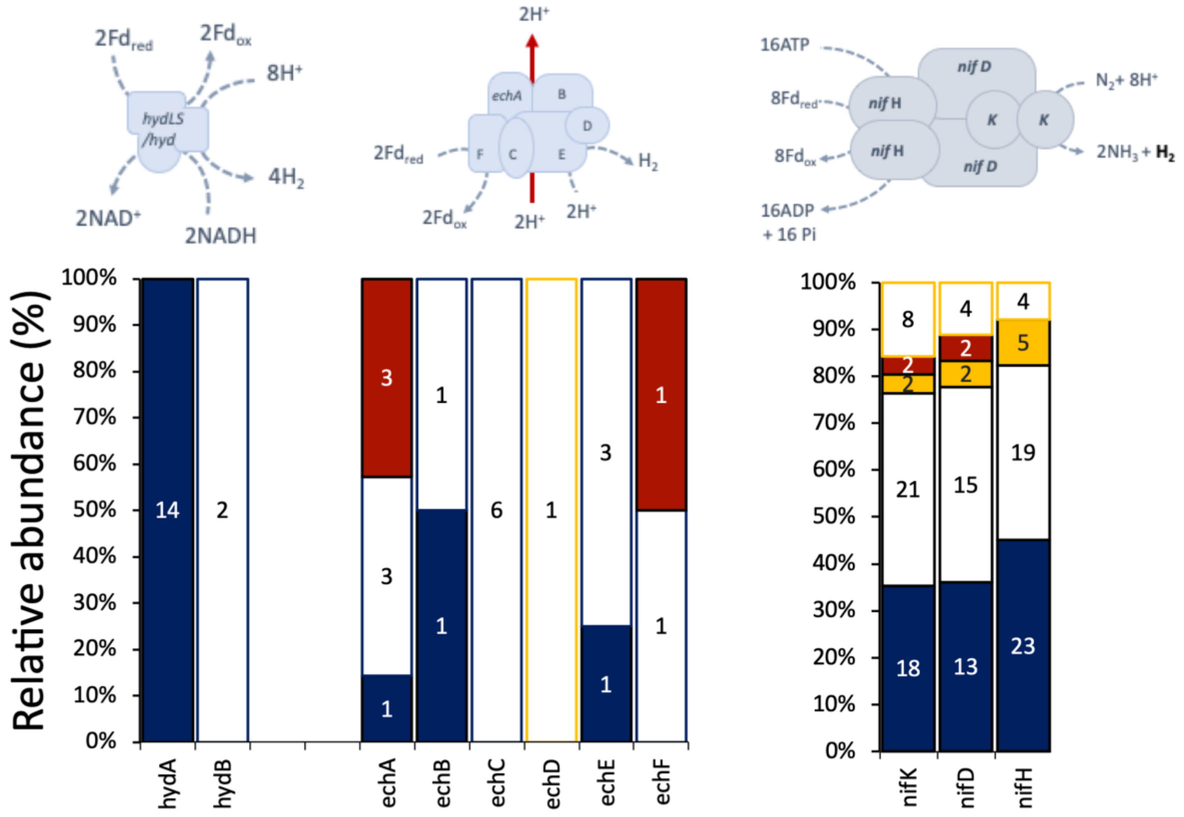

Figure 6S. Relative abundance of [FeFe]-ferredoxin-dependant hydrogenases which are H_2-evolving enzymes. Also, the relative abundance of nitrogenases in the metagenome of a corrosive community after 4 transfers on Fe^0. **Hyd-gene cluster EC:1.12.7.2**: H_2-evolving ferredoxin hydrogenases specific to Firmicutes fermenters and syntrophs; **Ech-gene cluster EC: 1.12.7.2**: energy converting hydrogenase involved in H2-evolution; diiron containing in Bacteria, nickel-iron containing in Archaea **Nif-gene cluster EC: 1.18.6.1**: molybdenum containing nitrogenase

Blue bars: Clostridia; empty blue bars: other bacteria; red bars: Methanosarcinales; Yellow bars: Methanobacteria; Empty yellow bars: other archaea;

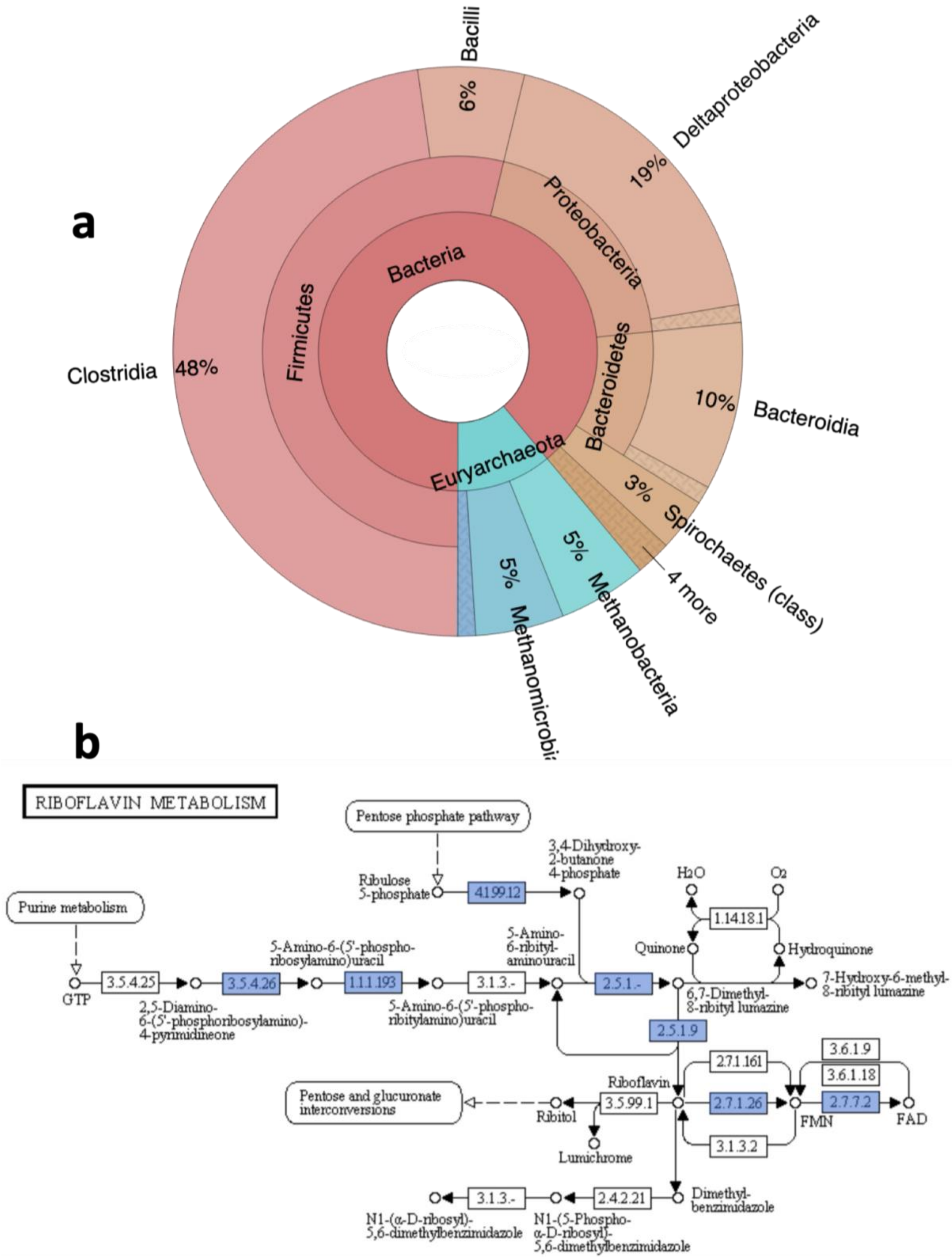

Figure 7S. Riboflavin biosynthesis (a) relative abundance of riboflavin biosynthesis genes in the metagenome of a corrosive community (b) KEGG map for the riboflavin biosynthesis pathway in *Clostridium*. All highlighted enzymes (in blue) could be discovered in the metagenome.
